# Identification of Potentially Novel Molecular Targets of Endometrial Cancer Using a Non-Biased Proteomic Approach

**DOI:** 10.3390/cancers15184665

**Published:** 2023-09-21

**Authors:** Anthony H. Taylor, Justin C. Konje, Thangesweran Ayakannu

**Affiliations:** 1 Reproductive Sciences Section, Department of Cancer Studies & Molecular Medicine, University of Leicester, Leicester LE1 7RH, UK; aht13@leicester.ac.uk (A.H.T.); jck4@leicester.ac.uk (J.C.K.); 2 Department of Molecular and Cell Biology, University of Leicester, Leicester LE1 7RH, UK; 3 Department of Health Sciences, University of Leicester, Leicester LE1 7RH, UK; 4 Weill Cornell Medicine-Qatar, Al Rayyan, Doha P.O. Box 24144, Qatar; 5 Department of Obstetrics & Gynaecology, Taylor’s University, Subang Jaya 47500, Selangor, Malaysia; 6 Sunway Medical Centre, Bandar Sunway, Subang Jaya 47500, Selangor, Malaysia

**Keywords:** biomarker, endometrial cancer, proteomics, protein, prognosis, therapy

## Abstract

**Simple Summary:**

The number of women diagnosed and dying from endometrial cancer worldwide continues to rise. The reason for these sad statistics is because unbiased early detection methods are lacking. Many molecular markers have been used to diagnose and characterise the different forms of endometrial cancer. Although some protein markers are used in this way, they are not good enough to help those subsequently identified as having endometrial cancer, because many of the proteins that are used to diagnose endometrial cancer are also found in patients with other forms of cancer. In this study, we have looked for, and found, new protein markers that are “unique” to endometrial cancer that can be used to better distinguish patients with endometrial cancer from patients with other diseases and also provide novel research targets for future treatment and cure for the ever-increasing endometrial cancer populations.

**Abstract:**

The present study was aimed at identifying novel proteins in endometrial cancer (EC), employing proteomic analysis of tissues obtained after surgery. A differential MS-based proteomic analysis was conducted from whole tissues dissected from biopsies from post-menopausal women, histologically confirmed as endometrial cancer (two endometrioid and two serous; n = 4) or normal atrophic endometrium (n = 4), providing 888 differentially expressed proteins with 246 of these previously documented elsewhere as expressed in EC and 372 proteins not previously demonstrated to be expressed in EC but associated with other types of cancer. Additionally, 33 proteins not recorded previously in PubMed as being expressed in any forms of cancer were also identified, with only 26 of these proteins having a publication associated with their expression patterns or putative functions. The putative functions of the 26 proteins (GRN, APP, HEXA, CST3, CAD, QARS, SIAE, WARS, MYH8, CLTB, GOLIM4, SCARB2, BOD1L1, C14orf142, C9orf142, CCDC13, CNPY4, FAM169A, HN1L, PIGT, PLCL1, PMFBP1, SARS2, SCPEP1, SLC25A24 and ZC3H4) in other tissues point towards and provide a basis for further investigation of these previously unrecognised novel EC proteins. The developmental biology, disease, extracellular matrix, homeostatic, immune, metabolic (both RNA and protein), programmed cell death, signal transduction, molecular transport, transcriptional networks and as yet uncharacterised pathways indicate that these proteins are potentially involved in endometrial carcinogenesis and thus may be important in EC diagnosis, prognostication and treatment and thus are worthy of further investigation.

## 1. Introduction

The number of patients with endometrial carcinoma (EC) at an advanced stage or of high histological grade is increasing, and prognosis has not improved over the last decade [1], even though EC is the most common gynaecological malignancy in the United States [2] and the fourth most common cancer in women elsewhere in the world, including Europe [3]. Most patients with low-grade or early stage EC are treated early and thus have a good prognosis [2,4]. Nevertheless, the incidence and mortality rates for those with advanced disease, especially non-endometrioid cancers (which are generally more clinically aggressive [5,6]), continue to rise [7,8]. The increase in EC incidence is due partly to a combination of factors such as increasing rates of obesity, metabolic syndromes (hypertension, diabetes mellitus type 2 and thyroid disorders), increasing lifespan, early menstruation, late menopause, tamoxifen use and high circulating oestrogen levels through local tissue conversion of steroid precursors [7,8,9,10]. Unbelievably, there has been no statistical improvement in mortality rates for high-grade, recurrent, metastatic disease, advanced disease and non-endometrioid EC [11] over the past 35 years [11]. The detection, especially of early stage EC, relies on the presence of common symptoms, which are not always evident in all patients [5,12], and globally, diagnosis relies on histological classification, as definitive lipid [13] or protein [1] biomarkers are currently unavailable. Furthermore, difficulties at identifying relapsed or late-stage ECs are associated with high morbidity and mortality [14]. The identification and characterisation of sensitive and specific biomarkers might help reduce late detection and mortality especially for advanced EC, improve risk assessment, facilitate screening and enable better choice of treatment [15]. Attempts to find such a set of novel protein biomarkers has provided conflicting data [16,17,18,19,20,21,22,23,24,25,26], because (1) the heterogeneity of EC is often not taken into account, with large sample sets reflecting that larger patient heterogeneity, (2) variations in study design, techniques and methodologies, cut-off values or overzealous probabilities make inter-study comparisons and validation challenging, (3) a lack of standardisation in sample procurement and processing introduces additional variability, (4) disparities in patient demographics, medical histories or hormonal status or treatments have often led to inconsistent biomarker associations and (5) publication bias, whereby positive (high expression) results tend to be published more frequently than negative or inconclusive findings, leads to an overrepresentation of unpromising or potentially misleading biomarkers, skewing perceptions of their true utility. To counteract these considerations, we have limited our patient pool to the most common forms of EC (endometrioid and serous EC), standardised patient demographics and our sample procurement and processing methodologies, have selected a cut-off change of >1.5-fold protein expression increase (as recommended by others [27]) and eliminated previously identified EC biomarkers already discovered to be present in other forms of cancer and thus removed the “general cancer bias” of previous discoveries. Previous inconsistencies in the literature led us to the conclusion that there is thus an urgent need for the discovery of novel molecular targets for EC diagnosis, prognosis and treatment, which will have the potential to improve clinical outcomes for EC patients. Our aim here was to identify such novel proteins in EC tissue using an unbiased proteomic approach that would aid early diagnosis. In doing so, we identified 33 functional proteins that appear to be uniquely expressed only in EC tissue. Of these, 26 have attendant literature that supports the notion that some of these proteins could be potentially involved in various cellular processes, such as metabolism, cell signalling and immune responses, which may contribute to EC development and progression.

## 2. Materials and Methods

### 2.1. Patients and Sample Collection

Women undergoing hysterectomy for either endometrial carcinoma (EC, cancer group) or a benign gynaecological condition, such as uterine prolapse (control/atrophic group) at the University Hospitals of Leicester National Health Service Trust were recruited. All provided signed written informed consent to take part in this study, which had Ethical approval from the Leicestershire, Northampton and Rutland Research Ethics Committee (LREC number 06/Q2501/49). Exclusion criteria were those currently on or had been on hormonal treatment (such as hormone replacement therapy or the levonorgestrel intrauterine system) or currently on prescription or recreational drugs. Women with any other type of cancer were also excluded. Fresh uteri obtained at hysterectomy were immediately transported on ice to the histopathology department where endometrial biopsies free of myometrium and other tissues were obtained by a consultant gynaecological histopathologist [28]. The biopsies were divided into two; one for the measurement of proteins within this study and the other for histological confirmation of the diagnosis, either EC or atrophic histologically normal tissue. Both cancer and normal endometrium biopsies were washed with phosphate-buffered saline (PBS) to remove excess blood and thereafter immediately stored either at −80 °C for protein extraction or in 10% formalin for histological confirmation of the diagnosis. Histological confirmation of the disease was kindly performed by the Hospital’s senior gynaecological pathology consultant and oestrogen/progesterone receptor status determined using standard immunohistochemical procedures. The age and BMI of each patient were also recorded and statistical differences between the groups determined using unpaired Student’s *t*-test (GraphPad Prism version 7.00 for Windows, GraphPad Software, La Jolla, CA, USA, www.graphpad.com).

### 2.2. Protein Preparation

Proteins were prepared as previously described [29]. Briefly, each endometrial biopsy (~25 mg) was taken from the freezer and defrosted directly in lysis solution (4% SDS, 100 mM Tris-HCl pH 7.6, 0.1 M DTT; 150 μL) and homogenised with a TissueRuptor (QIagen, Crawley, UK) at medium speed in 3 × 30 s bursts and on ice. The homogenate was then heated at 95 °C for 3 min to lyse the tissue. The lysate was then sonicated for 1 min to shear DNA and then clarified by centrifugation at 16,000× *g* for 5 min. Proteins (30 μL) were mixed with 200 μL of UA denaturing solution (8 M urea in 0.1 M Tris-HCl, pH 8.5) and placed onto a Microcon centrifugal filter unit (Millipore, Watford, UK) and centrifuged at 14,000× *g* for 15 min. The column was washed with 200 μL of UA solution (14,000× *g* for 15 min) and the flow through discarded. Iodoacetamide solution (0.05 M in UA solution, 100 μL) was added to the column and allowed to mix at 600 rpm in a thermo-mixer (20 °C) for 1 min before being incubated without mixing for an additional 20 min. The column was centrifuged at 14,000× *g* for 10 min. The column was washed 3 times with 100 μL of UA solution (14,000× *g* for 15 min each) and the flow through discarded. The protein mixture was then neutralised with ammonium bicarbonate (ABC) solution (0.05 M; 100 μL) and the pH made alkali with two more additions of ABC solution (14,000× *g* for 10 min). Then, 40 μL of trypsin (Sigma, Poole, UK) solution (0.4 mg/mL) was added and mixed at 600 rpm in the thermo-mixer (20 °C) for 1 min before being placed at 37 °C in a wet chamber overnight [30]. After centrifugation (14,000× *g* for 10 min), the columns were washed through with 40 μL of ABC solution to collect the trypsin digest. The effluent was acidified with an equal volume of 0.1% tri-fluoroacetic acid (Sigma) to stop the trypsin digestion. A 1 in 10 dilution of the sample was then used to quantify the protein/peptide concentration spectrophotometrically at 280 nm. The samples were then transferred to the Protein and Nucleic Acid Chemistry Laboratory (PNACL, University of Leicester, Leicester, UK), where the peptides were separated by reverse-phase chromatography and subjected to tandem mass spectrometry [29].

### 2.3. Sample Separation and MS/MS Analysis

The resulting peptide mixtures were loaded at a high flow rate onto a reverse-phase trapping column (0.3 mm i.d. × 1 mm), containing 5 μm C18 300 Å Acclaim PepMap media (Dionex, Camberley, UK) and eluted through a reverse-phase capillary column (75 μm i.d. × 150 mm) containing Jupiter Proteo 4 μm 90 Å media (Phenomenex, Macclesfield, UK) that were self-packed using a high-pressure packing device (Proxeon Biosystems, Odense, Denmark). The output from the column was directly sprayed into the nanospray ion source of a 4000 Q-Trap MS (Applied Biosystems, Warrington, UK). The analysis was carried out in the positive ion mode using data-dependent switching, as described in [29,31]. De novo sequencing (http://www.ionsource.com/tutorial/DeNovo/DeNovoTOC.htm; accessed on 6 September 2023) was carried out on the most intense fragment ion spectra using the BioAnalyst peptide sequencing tool (Applied Biosystems), and the outputted peptide fragment data were subjected to further in silico analyses. Each sample was processed twice, and the outputs combined to create a single output file for each patient biopsy.

### 2.4. In Silico Analysis of Peptide Fragments

Scaffold Proteome analysis software (version 4.11.0, Proteome Software Inc., Portland, OR, USA, http://www.proteomesoftware.com/, last accessed on 6 September 2023) was used to validate MS/MS-based peptide and protein identifications based on the Uniprot database. Peptide identifications were accepted if they could be established at greater than 95.0% probability. Peptide Probabilities from Mascot were assigned by the Scaffold Local false discovery rate (FDR) algorithm. The FDR for the fractionated peptide samples was 0.06%, resulting in an FDR for protein discovery at only 0.4% of the total identifiable proteins. Peptide Probabilities from X! Tandem were assigned by the Peptide Prophet algorithm [32] with Scaffold delta-mass correction. Protein identifications were accepted if they could be established at greater than 95.0% probability and contained at least two identified peptides and exceeded 10 ppm. Proteins that contained similar peptides and could not be differentiated based on MS/MS analysis alone were grouped to satisfy the principles of parsimony. Proteins sharing significant peptide evidence were grouped into clusters. X! Tandem and Mascot were used in subsequent analyses to identify common and previously undiscovered proteins in the 8 biological samples from a database of 40322 possible known proteins contained within the UniprotHuman_2013_08 FASTA Database. Data from these analyses were subjected to more detailed pathway/network analysis using the Reactome^®^ pathway database (version 85; https://reactome.org/ [33]; accessed on 6 September 2023) and PantherGO classification system version 15.0 (released 14 February 2020) that contains 15,702 protein families divided into 123,989 functionally distinct protein subfamilies (http://pantherdb.org/webservices/go/overrep.jsp; accessed on 6 September 2023). Data from these analyses established that proteins and pathways confined to atrophic tissues, exclusive to EC tissues and common to both sets of samples, exist. Data from these tables were examined and compared and contrasted with existing published data on the proteins expressed in human EC tissues, proteins not known to be associated with EC but associated with other forms of cancer (mainly ovarian, cervical, colorectal, breast, lung or haematological), or proteins not known to be associated with any form of cancer. Focused attention was placed on proteins associated with the networks identified by Reactome^®^ pathway/network analysis for the proteins only found in the EC tissue. In addition, these data outputs were examined for protein expression only in the Type 1 EC samples, only in the Type 2 EC samples or in both sets of samples (Supplemental Tables S1–S3). Furthermore, confirmation of protein expression uniqueness was determined by analysis of the TCGA cross-platform expression data available via the cBioPortal database (https://www.cbioportal.org, accessed on 6 September 2023). Further analysis was extended to the genome and transcriptome for gene alterations and mRNA expressions, respectively, of previously non-identified EC-specific proteins we report herein.

## 3. Results

### 3.1. Patient Characteristics

All eight patients (four normal and four with EC) were post-menopausal and aged between 55 and 88 years (Table 1). The mean age (±SD) was 60.0 ± 4.8 years for those with an atrophic (normal) endometrium and was 71.0 ± 13.6 years (*p* = 0.188) for those with EC. Those with cancer had a statistically higher BMI than those with atrophic endometrium (36.0 ± 1.6 kg/m^2^ versus 22.8 ± 3.0 kg/m^2^; *p* = 0.041), in keeping with the known prevalence of obesity in patients with EC [7]. Endometrial cancer biopsies were classified according to the revised FIGO classification system [34]. Prior to surgery, none of the patients received any hormonal treatments.

### 3.2. Proteins Expressed Only in Endometrial Cancer

**Figure 1:**
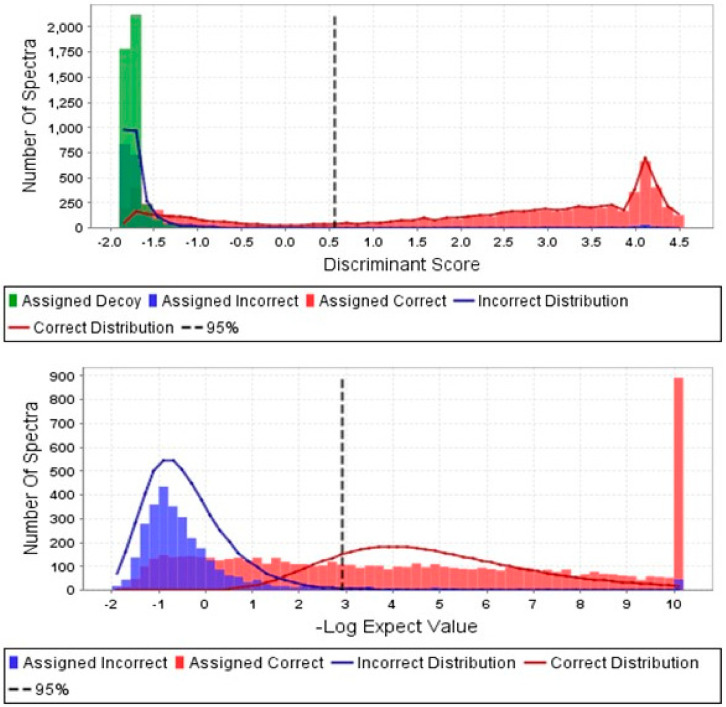
Scaffold Proteome analyses for correct spectral identification. The upper panel shows the number of peptide spectra assigned correctly (pink bars) compared to number of peptide spectra assigned incorrectly (green bars) together with their relative distributions (red and blue lines). The lower panel shows the log-transformed data for the number of spectra plotted against the negative log of the expected value of spectra based on the discriminant score shown in Panel A. Only 1 spectral line is a higher level than expected and that associates with serum albumin in both the control and cancer samples. The software can be accessed from www.proteomesoftware.com, last accessed 6 September 2023.

**Figure 2:**
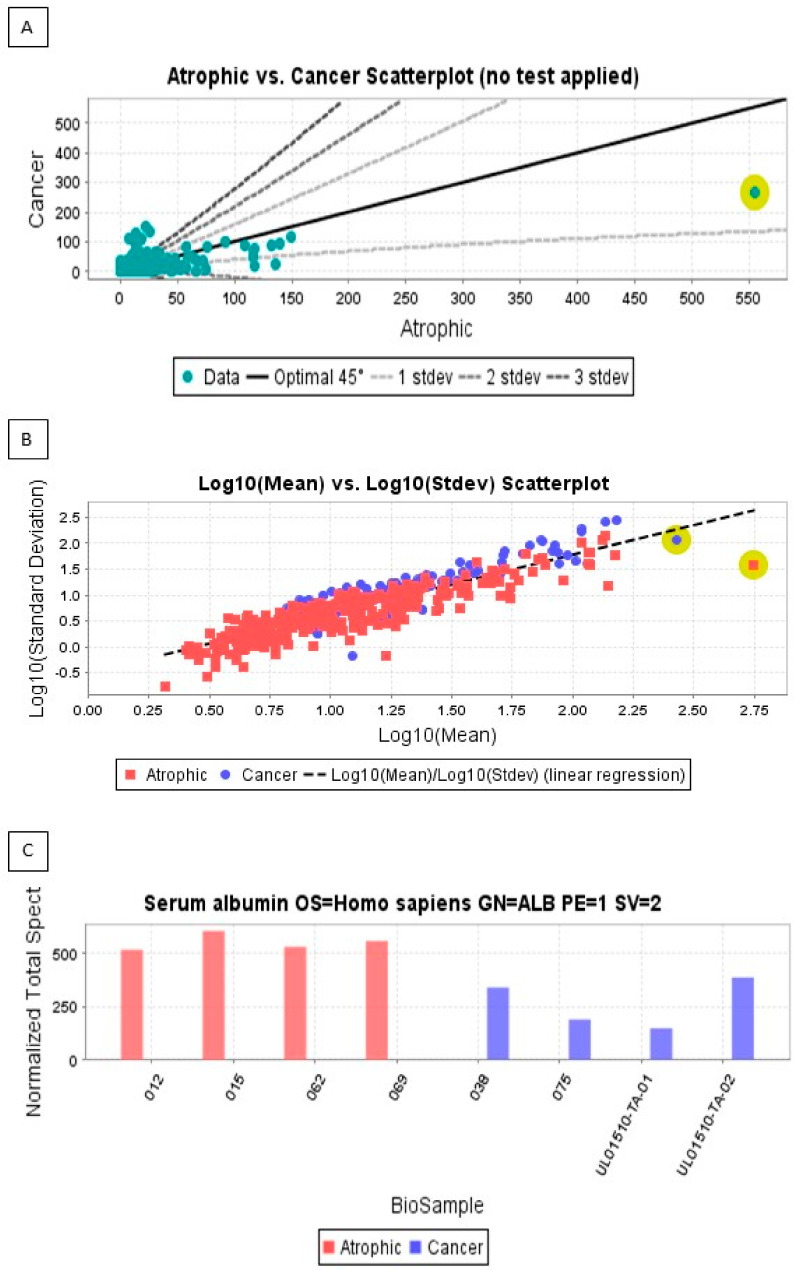
Scatterplot for the relative quantities of proteins identified in 4 control (atrophic) and 4 endometrial cancer (cancer) samples. The upper panel (**A**) shows the raw data, whilst the middle panel (**B**) shows the log-transformed data once the proteins outside the 3 standard deviations had been omitted. The protein highlighted with a yellow circle is serum albumin and was the most abundant protein in the atrophic samples (the data point for the cancer sample is outside the 3 standard deviations range). The spread of data is consistent with a high probability of protein identification. The lower panel (**C**) shows a histogram of positive peptide sequences identified in the biological samples. The data indicate the number of positive peptide sequences identified in the individual biological samples (control/atrophic: pink bars) and (endometrial cancer/cancer: blue bars) for the protein with the most abundant peptide spectra—serum albumin (highlighted in panel A).

A total of 2580 proteins were identified from 57431 individual peptide fragments across all samples. Of these, 706 and 666 proteins with > 95% probability were identified across the four atrophic and EC samples, respectively. The proportion of proteins that were incorrectly identified was 0.06% (Figure 1). Of the correctly identified proteins, those outside a threshold of the three standard deviations were excluded from subsequent analyses, leaving a total of 888 for subsequent analyses (Figure 2A,B). Logarithmic transformation (of the standard deviation for the peptide sequence expression level for the atrophic and cancer peptide fragments against the logarithmic transformation of the mean peptide sequence expression levels) indicated concordance with a linear model predictive of good quality data, with the most expressed protein being serum albumin for all samples (yellow dots in Figure 2B), despite copious washing of samples with saline prior to protein fragmentation. To confirm good adherence of signal and protein identification (Figure 2C) the most over-represented peptide spectra resulting in an over-representation of a single protein (serum albumin) were used to verify the entire dataset. The greatest variability was found in the four cancer samples. The most up-regulated proteins identified as being not only present in the EC samples but also in the atrophic endometrial control tissues included many gene/protein combinations previously identified elsewhere [23,35,36,37,38,39].

**Table 1:** Patient characteristics, biopsy description and pre- and post-operative therapy.

Patient ^†^	Age (yr)	BMI (kg/m^2^)	Medical Conditions ^1^	Sample Type ^2^	Cancer Grade	Cancer Stage	LVSI ^3^	Myometrial Invasion	Adjuvant Treatment	ER/PR Status ^4^
012	55	19	HTN	A	n/a	n/a	n/a	n/a	n/a	Nil
015	65	22	DM2	A	n/a	n/a	n/a	n/a	n/a	Nil
062	63	26	HTN/DM2	A	n/a	n/a	n/a	n/a	n/a	Nil
069	57	24	HTN	A	n/a	n/a	n/a	n/a	n/a	Nil
038	88	24	HTN	E	1	1	-ve	<50%	Nil, surgical follow-up for 3 years	ER + ve, PR − ve
075	74	42	DM2	E	1	1	-ve	<50%	Nil, surgical follow up for 3 years	ER + ve, PR − ve
TA-01	61	38	HTN	S	High grade	1	+ve	>50%	Chemo-radiation, surgical follow up for 3 years	ER − ve, PR − ve
TA-02	61	40	DM2	S	High grade	1	+ve	>50%	Chemo-radiation, surgical follow up for 3 years	ER − ve, PR − ve

^†^ Patient id here corresponds to sample labelling in Figure 2; ^1^ HTN = hypertension; DM2 = type 2 diabetes mellitus; ^2^ A = atrophic; E = endometrioid; S = serous tissue; ^3^ LVSI = lymphovascular space invasion; ^4^ ER + ve/ER − ve = oestrogen receptor positive/negative; PR + ve/PR − ve = progesterone receptor positive/negative; n/a = not applicable. All patients were post-menopausal.

**Figure 3:**
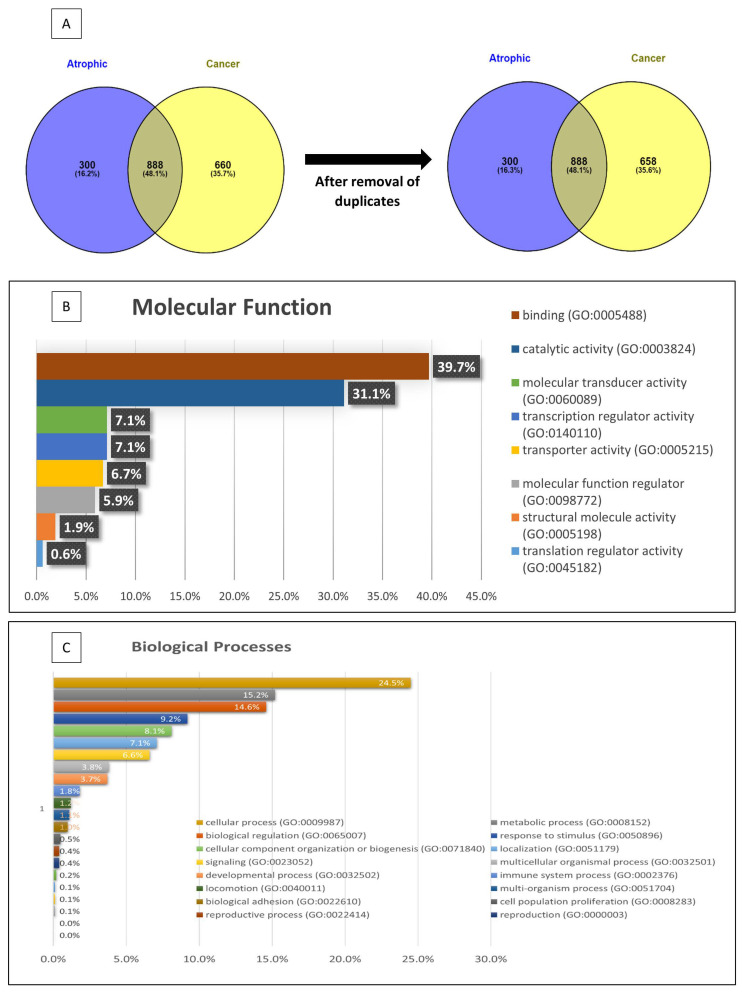
Venn diagram and bar charts for proteins identified using X! Tandem and Mascot. Venn diagrams (**A**) are used to show that a total of 1851 proteins were originally identified, of which 888 were common to both control (atrophic) and endometrial cancer (cancer) samples. Only 300 proteins were found solely in the control tissues, and 663 were only found in the cancer tissues. After close examination of the peptide identities, 4 proteins were duplicated in the cancer tissues and so were eliminated from further analyses (second Venn diagram). Gene ontology (GO) pathway analysis of the 663 proteins found only in endometrial cancer matched the genes identified to 21 biological processes (**B**) and to 8 molecular functions (**C**). The % of genes in each category is indicated.

**Figure 4:**
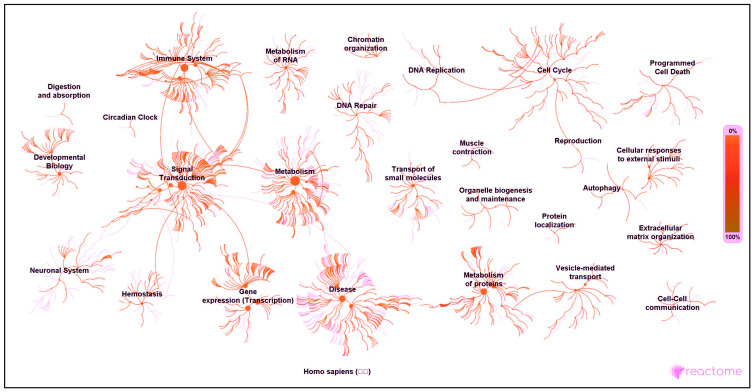
Functional analysis of cancer-specific protein pathways and interactions. The diagram depicts functional densities for the proteins identified as being exclusive to endometrial cancer. The data output is direct from the Reactome application. Note the enrichment of proteins in signal transduction, cell cycle, programmed cell death, metabolism, disease processes and reproduction. Also note that two distinct networks are prevalent: one on the left-hand side of the figure and focused on signal transduction and another on the right-hand side focused on the cell cycle.

**Figure 5:**
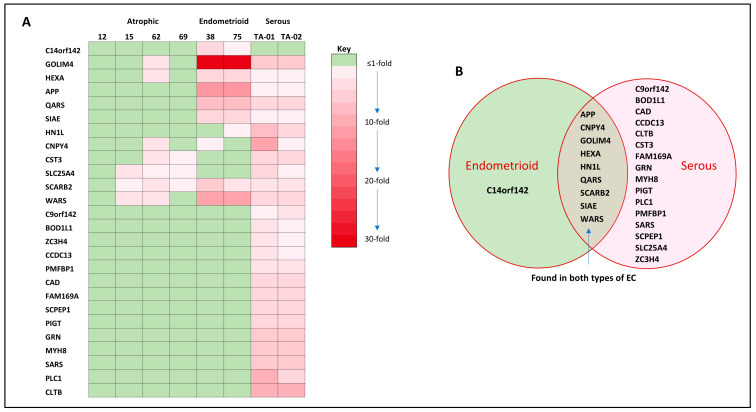
Expression of proteins unique to endometrial cancer. Panel (**A**) shows an expression heatmap for the 26 proteins identified to be uniquely expressed in EC. The fold-expression is related to the mean number of peptide spectra of the atrophic samples. When no peptide signal was identified in the atrophic samples, the expression in the serous samples was relative to the mean of all peptide spectra in the 4 atrophic controls. Panel (**B**) shows a list of proteins found only in the endometrioid (Type 1 EC) samples, serous (Type 2 EC) samples or both types of EC.

The proteins identified were further characterised by Mascot^®^ into those found in either normal (atrophic) tissue, EC tissue or both (Figure 3). Of the 1848 proteins identified, 888 were common to both normal (atrophic) and endometrial cancer samples (Figure 3A), with 300 being from normal (atrophic) tissue proteins and 660 confined to the cancer tissues (first Venn diagram, Figure 3A). Further detailed analyses of these data indicated that multiple copies of some proteins were identified during the curating of the peptide sequences, resulting in a final tally of 658 EC-specific proteins (genes) being available for pathway and network analyses (second Venn diagram, Figure 3A). From the 888 proteins common to atrophic and EC samples, a total of 487 EC up-regulated proteins with a fold-change difference between the groups of at least 1.7 were identified. The top 100 proteins are listed in Supplemental Table S1. Of particular interest is the 2.5-fold up-regulation in EC of HE4 (also known as WFDC2), which is now used in some centres to help in the diagnosis of EC (Supplemental Table S2) [40,41,42,43]. The majority (67%) of the up-regulated proteins were in both types of EC, with 9% up-regulated only in Type 1 EC and 24% up-regulated only in Type 2 EC. In the top 100 up-regulated proteins (Supplemental Table S1), none were up-regulated only in Type 1 EC and two proteins (nucleolin and the beta subunit of glucosidase 2) were up-regulated only in Type 2 EC, whilst the remaining 97 proteins were up-regulated in both Types of EC, suggesting that common oncogenic pathways operate in both forms of EC. However, the list is incomplete, and many other proteins (788 in total) that were present in the atrophic samples and up-regulated in EC are not shown because they either fell outside the expression range (below the cut-off threshold) or were absent from the atrophic endometrial samples but were present in the EC samples and thus demonstrated an infinite fold-change in expression. In order to identify novel EC protein signatures, these proteins were subjected to pathway and network analyses (Figure 4), and those proteins already known to be up-regulated in EC (Supplemental Table S2) or known to be expressed in other forms of cancer (Supplemental Table S3) were eliminated from further consideration. These manipulations produced a list of 33 proteins, of which only 26 have any supporting literature (Figure 5). The data indicated that only one protein, C14orf142, unique to endometrioid (Type 1) EC was identified in this way, whilst 16 proteins (C9orf142, BOD1L1, CAD, CCDC13, CLTB, CST3, FAM169A, GRN, MYH8, PIGT, PLC1, PMFBP1, SARS, SCPEP1, SLC25A4 and ZC3H4) were shown to be uniquely identifiable in serous (Type 2) EC (Figure 5A). By contrast, nine proteins (APP, CNPY4, GOLIM4, HEXA, HN1L, QARS, SCARB2, SIAE and WARS) were shown to be only present or up-regulated in both types of EC (Figure 5B) and so may be of particular importance for future study.

### 3.3. Pathway and Network Analyses

Panther gene ontology (GO) pathway analysis of the 658 EC proteins matched them to 21 biological processes (Figure 3B) and 8 molecular functions (Figure 3C). The proteins identified in each of these processes and functions are listed in Supplemental Tables S1–S3. The key biological processes were cellular in origin, metabolic, regulatory or in response to stimuli (Figure 3B). These four biological processes constituted 23.8% of the proteome changes in EC. Further analysis of the functions of these proteins at the molecular level indicated that 70.8% of the proteins are involved in binding or catalytic activities (Figure 3C), suggesting that during EC cell formation and stability, ligand binding and enzymatic changes occur and are necessary and important for cancer cell survival. Networks between different proteins identified in the EC pathway were subjected to Reactome^®^ pathway analysis, which indicated that proteins in multiple functional pathways were affected in EC (Figure 4). In this analysis, only significantly expressed proteins (two-way ANOVA; *p* < 0.01) were included in the pathway overview diagram (Figure 4) and subsequent tables (Supplemental Tables S1–S3). Each protein had to be identifiable with a minimum of two peptides, and the intensity of the colour and size of node in the centre of each event indicated the importance of that event. Thus, the key functional pathways of the 27 different networks listed were identified as being inflammation/immune system, protein metabolism, gene expression, disease and oxidative processes, although other networks were identified. Signal transduction appeared to be central to these networks, with that network interacting with the immune system, metabolism, gene expression (transcription), haemostasis and the neuronal system (Figure 4, left-hand side). An additional network was identified with the cell cycle at its centre and linking DNA replication to programmed cell death, cellular responses to be to reproduction through an alternative pathway (Figure 4, right-hand side), whilst no proteins were identified in the digestion and absorption network (top left). The Reactome^®^ pathways and networks were also used to characterise the proteins that have previously been identified or associated with EC, not known in EC but associated with other forms of cancer or not reported to be associated with any form of cancer (Supplemental Tables S1–S3).

In each case, several proteins were mapped to more than one single network or pathway, indicating that different pathways may be simultaneously affected in EC. The only network that did not contain any EC proteins was the digestion and absorption network (Figure 4 and Supplemental Tables S2 and S3). In each case/protein, we identified a representative publication for each protein listed in this study for the interested reader, and this is supplied in Supplemental Tables S2 and S3. The data show that of these “cancer” proteins, i.e., potential markers of several different forms of cancer (including EC), 138 (10%) were associated with only Type 1 EC, 472 (40%) were associated with Type 2 EC and 669 (50%) were found in both types of EC, indicating that the tissue under investigation had many proteins in common with other forms of cancer. Many of the proteins may be key regulators of several important networks since they appear several times in multiple cellular and biological processes. Nevertheless, the aim of this study was to identify novel proteins that have not been reported in any forms of cancer.

Of the identified proteins (Table 2, Figure 5) that have not been previously associated with EC or any other cancer types (i.e., APP, BOD1L1, CAD, CCDC13, CLTB, CNPY4, COPE, CST3, C9orf142, c14orf142, DKFZp313H139, E7EPZ9-DECOY, FAM169A, GOLIM4, GRN, HEXA, HN1L, IGKV-I, IGKV-II, IGLV-III, J3QQ66-DECOY, MYH8, PIGT, PLCL1, PMFBP1, PPM1G, PPP1R2, QARS, Q8WZ42-2-DECOY, Q8WZ42-12-DECOY, SARS2, SCARB2, SCPEP1, SIAE, SLC25A24, WARS and ZC3H4), 12 proteins (DKFZp313H139, COPE, CCDC13, CAD, APP, CLTB (protein fragments or parts of existing proteins or fusion proteins); IGKV-I, IGKV-II, IGLV-III (uncharacterised immunoglobulin chains); and E7EPZ9-DECOY, Q8WZ42-12-DECOY and Q8WZ42-2-DECOY (peptide fragments used in pharmaceuticals)) were found to not have any known association with any biological function or cellular process and thus have no attendant literature source to draw upon. Nevertheless, what little information is known about these proteins is presented in the Supplemental Information File. The remaining proteins have some literature available, and a link to the relevant literature is provided (Table 2) and presented as information with some commentary within the Supplemental Information File. The 33 proteins unique to EC were mapped to processes known to have key roles in the development of cancer, such as developmental biology, disease processes, extracellular matrix organisation, transcription and ribosomal metabolism, programmed cell death and vesicle transport (Table 2). Interestingly, some of these proteins are mapped to muscle contractility, suggesting that intracellular contraction machinery is available for endometrial cancer cell movement, a key component of metastatic disease. Nevertheless, 21 of the identified proteins did not map to any known pathways or networks, suggesting that very little is known about these particular proteins. What is known is presented in the Supplemental Information File.

## 4. Discussion

With our innovative non-biased approach, we identified 1846 protein signatures, of which 300 were only found in normal atrophic endometrium and 658 were unique to EC. A total of 888 proteins were common to both types of tissues and modulated in EC. Of these, 487 had at least a 1.7-fold increase in expression when compared to normal atrophic endometria, and the top 100 proteins are presented in Supplemental Table S1. These data are qualitatively similar to those reported by others [16,17,18,19,20,21,22,23,24,25,26]. A total of 658 proteins identified to be present in only EC were matched by pathway and network analysis to 21 biological processes (response to stimuli, metabolic, regulatory and cellular). Of these various proteins that mapped to important pathways and networks involved in cancer formation, 33 had not been previously associated with any type of cancer. Since these proteins are reported here for the first time, their usefulness as early diagnostic tools, prognostic markers or targets for the development of therapeutics can only be speculated upon but could provide the basis for further detailed research.

In the past 20 years, the examination of global protein expression via proteomics as a suitable strategy for cancer research discovery has accelerated, with global protein examination for potential new biomarkers [11,35,37,38,71,72,73,74,75,76,77,78,79,80,81]. Various proteomic methods for discovering potential EC biomarkers have used differential protein screening using cancerous and non-cancerous endometrial tissues [11,35,37,38,39,82,83,84,85,86,87,88] (also see Supplemental Table S1). An alternative “bottom-up” approach that involves chemical labelling of peptides resulting from enzymatic digestion of sample proteins, followed by mixing of the control and test samples prior to liquid chromatography–tandem mass spectrometry (LC-MS/MS) analysis (which ensures identical treatment of the study samples), has been one of the most extensively used technologies for cancer biomarker discovery [16,21,71,72]. Because tissue samples used in such studies are often complex mixtures, pre-fractionation of the samples using strong cation exchange (SCX) is typically performed prior to analytical separation on a nanoscale reverse-phase (RP) column. The output from such columns, which is coupled in-line to a mass spectrometer (MS), often resolves some of the complexity; however, there is still a tendency for many peptides to co-elute during the RP separation, despite this two-dimensional chromatographic separation. Therefore, in any automated data acquisition, the MS is typically only able to detect a fraction of the peptides (due to time constraints), with the MS software tending to favour analysis of the most abundant peptides at the expense of co-eluting peptides of lower abundances. This consideration is of critical importance because many cell signalling, regulatory proteins and many other cancer-relevant proteins are usually expressed in low concentrations and consequently result in this “bottom-up” and “shotgun” approach missing some of the most valuable information [89]. By contrast, an iterative analysis of the same sample, coupled with an exclusion list of identified peptides that is generated after each run and used to inform the choice of ions that are targeted for MS/MS analysis during subsequent runs, appears to be a possible solution to address this concern, since this strategy forces the MS to target new, less-abundant peptides for MS/MS analysis during each of the successive iterations. Variations of this “drill down approach” have been reported for matrix-assisted laser desorption/ionisation (MALDI)-MS/MS and ESI-MS/MS [74,90]. Using this tactic, recognised peptide ions and their retention times are used to generate an exclusion list after the first LC-MS/MS analysis (for further iterations of LC-MS/MS analysis), resulting in a reduction in identifiable peptides and a limited ability to facilitate the identification of potentially novel molecular targets for the diagnosis, prognosis and/or therapeutic intervention for EC. This approach is cumbersome and excludes peptides or proteins that are differentially regulated by small changes in expression, whereas a more inclusive approach might yield better results.

Our approach was conceptually different to other approaches in that we combined our EC outputs and identified those peptide sequences that are either only present in the control (atrophic) samples or in the endometrial cancer samples, with the emphasis on the latter goal. Our aim here was to identify novel proteins that have not previously been associated with EC and not repeat previously reported increased or decreased expression in EC when compared to a control tissue but rather identify those proteins that might be “unique” to EC. Although this approach is similar to the “selective precursor ion exclusion” (sel-PIE) method of Wang et al. [89], their method excluded the proteins that are expressed at low detection levels and increased those expressed at high levels in atrophic tissues and decreased levels of expression in cancer, because it only detects proteins present or absent in the EC samples regardless of the protein’s original expression level. To the best of our knowledge, this is the first time that a combination of protein quantification and an “all or nothing analysis approach” has been used for the identification of EC protein biomarkers. Nevertheless, this approach also identified proteins that are quantitatively and differentially up-regulated in endometrial cancer per se, so those data are also included in these analyses as a secondary aim of the study (Supplemental Table S2).

The tissues samples chosen were selected to generate a clean global differential profile of EC-specific proteins when compared to normal atrophic endometrium from patients that had similar characteristics and were very similar to those used by the TCGA consortium [26]. From these samples, we identified novel proteins that are unique to EC without using a biased selection approach that would identify proteins at a low peptide count or uniquely expressed in EC. By contrast, others have used various and disparate approaches. For example, De Douza et al. used multi-dimensional liquid chromatography and tandem mass spectrometry in a comparison of twenty Type 1 and Type 2 ECs with normal endometrium from twenty pre-menopausal women (10 secretory and 10 proliferative) [91]. Similarly, Byrjalsen et al. used two-dimensional gene analysis coupled with matrix-assisted laser desorption/ionisation time-of-flight (MALDI-TOF) mass spectrometry to evaluate five ECs, five endometrial hyperplasia and six normal endometria (five of which were proliferative) [92]. It has been suggested that the small number of tissue samples and less sensitive methods of proteomic analysis utilised in these studies (and herein) could have resulted in the limited identification of differentially expressed proteins [93]. We disagreed and instead generated a “clean” set of data that are less dependent on patient variability; as has been seen in previously mentioned studies, the data are therefore meaningful. Other potential causes of concern from previous studies include the effect of the menstrual cycle and the inclusion of pre-cancerous lesions in the sample collection making effective comparison and therefore conclusions from studies not using the same types of samples difficult. For these reasons, together with fundamental differences in sample selection and preparation, it is difficult to render an accurate comparison of the present study to these previous data; nevertheless, a deliberate attempt at comparison using limited patient numbers was made, as others have done [93]. The use of large sample numbers in proteomic studies of EC is resource-intensive, and so several studies have focused on the data obtained from the TCGA database. This strategy has several limitations including differences in protocols and the reliance on single datasets that results in disparate analyses and confusion in the literature. For these reasons, we used samples from histologically well-defined normal (atrophic) control tissues and ECs representing Type 1 EC and Type 2 EC. By doing so, we increased the probability of identifying novel EC marker proteins (for future study) and made our observations comparable (at the molecular level) to that of the TCGA consortium, who also used endometrioid (Type 1 EC) and serous (Type 2 EC) samples.

We deliberately ignored proteins that are present in normal atrophic endometrium but are increased in expression during the development of EC, because many of these proteins have previously been described (Supplemental Tables S1 and S2) or are found in other forms of cancer and hence are not specific to EC (Supplemental Table S3). It is also important to note that the novel EC proteins, APP, CAD, BRD2 (which is part of DKFZp313H139), CST3, GRN, PPM1G and ZC3H4 [94,95,96,97,98,99,100], have all been reported to be positively associated with obesity or adiposity [94,95,96,97,98,99,100], whilst the novel EC protein SLC25A24 may even prevent obesity and promote leanness [101]. This is important because obesity increases the risk of developing some forms of EC [102], and as such, studying these proteins may lead to a better understanding of the role of adiposity and obesity in the natural progression of these forms of EC.

Interestingly, 19 proteins in the metabolism of the RNA network identified by Reactome^®^ pathway analysis were found to be ribosomal proteins. Thirteen such proteins (RS3, RS9, RS14, RS18, RLA1, RLA2, RL8, RL11, RL18, RL22, RL24, RL10A and RL27A) have previously been described as associated with EC [93], but remarkably this was not confirmed when these gene names were inserted into PubMed, as they were not known to be linked with EC despite this having been already reported [93]. This shows a problem with curated databases in that the information they contain is often incomplete or inaccurate [103]. We identified previous investigations that reported differential expression of ribosomal proteins in breast [104], liver [105] and gastric [106] carcinoma compared to corresponding normal epithelial controls, suggesting that ribosomal protein expression is important in the development of numerous epithelial cancers, including those of the endometrium. Furthermore, recent advances in ribosomal research have indicated that ribosomal proteins have a variety of extra ribosomal functions (independent of protein biosynthesis) such as replication, DNA repair, inflammation, apoptosis and scavenging of reactive oxygen species [107]. This was exemplified by the cross-network interactions of several of these proteins with other biological functions (Supplemental Tables S2 and S3 and Figure 4).

Comparative analysis of different epithelial cancers indicates that overexpression of specific ribosomal proteins may be exclusively associated with specific tumour types and not necessarily a by-product of overall proliferation of these tumours [107,108]. Among the ribosomal proteins that we observed to be associated with our EC samples, RPS18 and RPL18A have been previously reported in association with colon cancer [109] and RPS14 and RPS18 in prostate cancer [109].

However, the preceding information was not the main focus of this study but to identify novel proteins that are expressed at an elevated level that are indicative of EC. To achieve that aim, we removed from our analyses all those proteins that had previously been identified to be associated with EC and set them to one side (Supplemental Table S2). In that group, we identified many of the proteins that were expected to be found that are associated with the cell cycle, developmental biology, disease, metabolism, programmed cell death, signal transduction and the transport of small molecules. In addition, we also identified proteins that are altered in EC that we and others have reported to be modulated in EC, such as CB1, CB2, GPR55, FAAH, NAPE-PLD, HE4, ESR1, PGR, WFDC2, etc. [1,17,28,38,42,110,111,112,113], although this list is not exhaustive. We then identified all the proteins that emanated from the mass spectrometer that have been associated with other forms of cancer (Supplemental Table S1) and removed those too from our analyses (Supplemental Table S3), since our goal was to identify novel EC proteins. After these exclusions, a total of 33 proteins remained that had not been previously identified as EC proteins. We used the available databases to mine for information about these proteins, some of which are discussed as to their possible role in EC (these data are presented in Supplemental Information File). The putative functions of those proteins centre around metabolism, inflammation and immune response and cell signalling functions that often become dysfunctional in EC or may contribute to cancer development and progression [7,114,115,116]. Whether these expression patterns are a cause or effect of EC requires further investigation but crucially are supported by data produced by the TCGA consortium (see limitations section below).

The data presented here therefore focused on identifying several proteins that are only present in the EC tissue and those that are differentially up-regulated in EC. When examined further, the proteins that are only found in the EC tissue fall into three categories: (i) those already known to be associated with EC; (ii) those already known to be associated with other forms of cancer but not EC; and (iii) those not previously linked with cancer at all. These three categorical EC-specific protein groups thus provide potential novel molecular targets for the diagnosis, prognostication and treatment of patients with EC. These laudable aims are beyond the scope of the present study but would be the goal of future research using the potentially novel proteins identified herein as a key starting point.

An explanation for the inability of the cBioPortal analyses to identify changes in protein expression, when transcriptome and genome data are excluded, could be due to the antibody array methodology used to generate the protein expression in that original study. That technique relies on the presence of an antigen-specific antibody being present on the array, and the lack of such antibodies or insufficient specificity due to post-translational modifications could explain why expression changes in the 33 unique EC proteins were not captured.

One limitation of our study is that our method did not include an analysis of phosphoproteins, lipoproteins or glycoproteins in EC, and this should be considered for future experimentation, since these could be important biomarker modifications, as has been recently demonstrated in numerous other cancer types including EC [36]. Additionally, we and others have not examined microRNA or short hairpin RNA expression, even though the presence of some of these may provide important starting points for future research, as they may provide functional evidence that some proteins may be reduced in expression [104], as we have reported for the cannabinoid receptors CBR1 and CBR2 [111]. Another key point of limitation is that EC samples from pre-menopausal women were not included, even though the incidence of EC in that patient cohort is increasing [117]. The reason for their exclusion from the present study was to reduce the impact that the menstrual cycle might have as a confounder of the protein expression patterns observed. An important supporting piece of evidence arising from this study, however, is that most of the proteins we report as being “unique” to EC have previously been demonstrated to have altered expression at the genomic and transcriptome level in EC but not at the protein expression level [26]. Thus, an important first next step for us was a clear verification that some of these proteins would make robust biomarkers of the disease, as has been stated previously [19]. To that end, we re-examined the genome, transcriptome and proteome data generated by the TCGA consortium [26] available through the cBioPortal access page (https://www.cbioportal.org, accessed on 6 September 2023).

When examining only protein expression, none of the 33 unique EC proteins were represented. When the same TCGA data were re-analysed with a transcriptomic query, 20 out of the 33 EC unique proteins had corresponding regulated mRNA expression in the Type 1 endometrioid EC and Type 2 serous EC samples. These data are qualitatively similar to our data, except the TCGA study used high-grade samples, whereas we used lower grade 1, stage 1 samples (Table 1), and it is possible that if early stage and grade samples were examined in the TGCA study (with a more comprehensive antibody array), their proteomic analyses may have been similar to what we report here with APP, CNPY4, GOLIM4, HEXA, HN1L, QARS, SCARB2, SIAE and WARS all appearing in that protein database.

Of the 20 transcriptional changes discovered, only mRNA levels for CAD and QARS were consistently up-regulated in 11% and 19% of the TCGA cases, respectively; all other genes either demonstrated increased and decreased mRNA levels, decreased levels or no alterations. This highlights one issue of focused research (such as examining proteomics data alone), in that additional genomic and transcriptomic information can be overlooked [103]. It also means that lipidomic, metabolomic and phospholipidomic data could also be missing, as was the case with the original TCGA study [91]. To compensate for the metabolomics issue, we deliberately took our protein outputs and tested them within the Reactome^®^ and MASCOT^®^ software packages, since these packages link directly to curated metabolomics databases [118,119]. This manipulation was used to provide initial functional considerations to the proteins we identified (see the Supplemental Information File).

## 5. Conclusions

The present study resulted in the identification of previously unknown proteins expressed in EC, all of which are potential clinical and biological markers of the disease. Some of these proteins offer potential targets within dysregulated signalling and enzymatic networks not previously described to be associated with EC that could be used not only in the understanding of EC pathogenesis but also in the development of novel therapeutic strategies and possible prognostic markers.

Accordingly, the novel proteomics data presented provide important starting points for numerous future investigations.

## Figures and Tables

**Table 2:** Proteins identified as not previously being associated with endometrial cancer or any other forms of cancer.

Reactome^®^ Network	Proteins Identified in Network *	PubMed ID (Where Available) **
Autophagy	There are no proteins to report in this category.	
Cell cycle	There are no proteins to report in this category.	
Cell–cell communication	There are no proteins to report in this category.	
Cellular responses to external stimuli	There are no proteins to report in this category.	
Chromatin organisation	There are no proteins to report in this category.	
Circadian clock	There are no proteins to report in this category.	
Developmental biology	CLTB ^2^; GRN ^2^	[44]; [45]
Digestion and absorption	There are no proteins to report in this category.	
Disease	APP ^B^; HEXA ^B^	[46]; [47]
DNA repair	There are no proteins to report in this category.	
DNA replication	There are no proteins to report in this category.	
Extracellular matrix organisation	APP ^B^	[46]
Gene expression (transcription)	GRN ^2^	[45]
Haemostasis	APP ^B^; GRN ^2^	[46]; [45]
Immune system	APP ^B^; CST3 ^2^; GRN ^2^; IGKV-I ^B^; IGKV-II ^B^; IGLV-III ^1^	[46]; [48]; [45]; no ref.; no ref.; no ref.
Metabolism	CAD ^2^; COPE ^1^; HEXA ^B^; QARS ^B^; SIAE ^B^	[49]; no ref.; [47]; [50]; [51]
Metabolism of protein	APP ^B^; COPE ^1^; CST3 ^2^; QARS ^B^; WARS ^B^	[46]; no ref.; [47]; [50]; [52]
Metabolism of RNA	QARS ^B^	[50]
Muscle contraction	CAD ^2^; GRN ^2^; MYH8 ^2^	[49]; [45]; [53]
Neuronal system	There are no proteins to report in this category.	
Organelle biogenesis and maintenance	There are no proteins to report in this category.	
Programmed cell death	CAD ^2^	[49]
Protein localisation	APP ^B^	[46]
Reproduction	There are no proteins to report in this category.	
Signal transduction	APP ^B^	[46]
Transport of small molecules	There are no proteins to report in this category.	
Vesicle-mediated transport	APP ^B^; CLTB ^2^; COPE ^1^; GOLIM4 ^B^; SCARB2 ^B^	[46]; [44]; no ref.; [54]; [55]
Found by GO pathway analysis but missing from Reactome^®^ network analysis	BOD1L1 ^2^; C14orf142 ^1^; C9orf142 ^2^; CCDC13 ^2^; CNPY4 ^B^; DKFZp313H139 ^2^; E7EPZ9-DECOY ^2^; FAM169A ^2^; HN1L ^B^; J3QQ66-DECOY ^2^; PIGT ^2^; PLCL1 ^2^; PMFBP1 ^2^; PPM1G ^B^; PPP1R2 ^B^; Q8WZ42-12-DECOY ^2^; Q8WZ42-2-DECOY ^2^; SARS2 ^2^; SCPEP1 ^2^; SLC25A24 ^2^; ZC3H4 ^2^	[56]; [57]; [58]; [59]; [60]; no ref.; no ref.; [61]; [54]; no ref.; [62]; [63]; [64]; [65]; [66]; no ref.; no ref.; [67]; [68]; [69]; [70]

* The EC type where protein expression is increased is denoted by the superscripted number or letter: Type 1 EC = 1, Type 2 EC = 2 and both types of EC = B; ** no ref = no suitable literature reference in the PubMed database.

## Data Availability

Data are available by request to the senior author.

## Supplementary Materials

The following supporting information can be downloaded at: https://www.mdpi.com/article/10.3390/cancers15184665/s1, Supplemental Table S1: The top 100 proteins differentially up-regulated in endometrial cancer relative to the level in atrophic endometrium; Supplemental Table S2: Proteins identified as already being associated with endometrial cancer; Supplemental Table S3: Proteins not previously associated with endometrial cancer BUT known to be associated with other forms of cancer; Supplemental Information File.
