# Supplementary material for: Identification of Potentially Novel Molecular Targets of Endometrial Cancer Using a Non-Biased Proteomic Approach
Source: Cancers (Basel). 2023 Sep 21;15(18):4665. doi: 10.3390/cancers15184665 (PMC10527058; doi:10.3390/cancers15184665)
Supplement: Supplementary file 1 [file cancers-15-04665-s001.zip › Supplemental Infromation file.pdf]

Supplemental Information. Putative functions of the proteins found to be unique to EC

Introduction

The proteins found to be unique to EC tissue within this study (**Table 2**) are presented alphabetically and their putative functions described and where applicable, briefly discussed. These functions are yet to be proven in EC, but create interesting starting points for future experimentation.

APP

APP (amyloid beta precursor protein), functions as a cell surface receptor and performs physiological functions on the surface of neurons relevant to neurite growth, neuronal adhesion and axonogenesis [1]. It captures divalent cations, and it is also involved in copper homeostasis/oxidative stress through copper ion reduction. *In-vitro*, copper-metallised APP induces neuronal death directly or is potentiated through Cu^{2+}-mediated low-density lipoprotein oxidation [2]. It can also regulate neurite outgrowth through binding to components of the extracellular matrix such as heparin and collagens I and IV, two proteins identified to be over expressed in this proteomic study (**Supplemental Table S1**). These data suggest that this protein is important in the development of EC through a number of disparate mechanisms yet to be examined.

BOD1L1

BOD1L1 (Biorientation of Chromosomes in Cell Division 1 like 1) is a nuclear protein that is predicted to enable protein phosphatase 2A binding activity and protein phosphatase inhibitor activity [3]. It has a putative involvement in a cell's response to DNA damage because it is a component of the fork protection machinery required to protect stalled/damaged replication forks from uncontrolled DNA2-dependent resection.

CAD

CAD (Carbamoyl-Phosphate Synthetase 2 or Aspartate Transcarbamylase) has 3 enzymatic activities needed in the 6-step pathway of pyrimidine biosynthesis. This trifunctional protein aids cell proliferation through *de novo* synthesis of pyrimidine nucleotides and uses the mitogen-activated protein kinase (MAPK) cascade to initiate this function [4], indicating a direct link between MAPK and pyrimidine nucleotide biosynthesis.

CCDC13

Very little is known about the function of CCDC13 (Coiled-Coil Domain-Containing Protein 13), apart from it is requirement for primary cilia formation where it promotes the localisation of the ciliopathy protein BBS4 to both centriolar satellites and cilia bodies [5]. In embryogenesis, cilia are known to be important in the correct movement of cells during tissue development and their failure is often associated with situs inversus [6]. It is therefore likely that CCD13 is involved in the movement or migration of EC cells and so could be a key component of metastatic EC disease.

CLTB

Clathrin light chain B (CTLB) is a smaller light chain component of the large, soluble protein clathrin that functions as the main structural component of the lattice-type cytoplasmic face of coated pits and vesicles which entrap specific macromolecules during receptor-mediated endocytosis [7]. The light chain version is implicated in cell spreading and migration [8] and so could be an important factor in metastatic EC disease.

CNPY4

CNPY4, also known as Canopy FGF Signalling Regulator 4, regulates the expression of the cell surface receptor TLR4 [9] a key regulator of immunomodulation that act in anti-tumour events. Recently, TLR4 has been identified as a key player in a regulatory pathway of aggressive malignancies whereby this protein activates inflammatory processes to attenuate tumour cell growth, especially in ovarian,

cervical, and ECs [10]. These data suggest that the increased expression of CNPY4 in our cohort of EC patients may be part of an early event to activate TLR4 and prevent tumour aggressiveness, even though TLR4 was not identified in the proteomic outputs.

COPE

COPE (Coatomer Protein Complex Subunit Epsilon) is a protein subunit of the coatomer complex. It is a cytosolic protein complex that binds to dilysine motifs and reversibly associates with Golgi non-clathrin-coated vesicles, which further mediate biosynthetic protein transport from the ER, via the Golgi up to the trans Golgi network [11]. Since the coatomer complex is required for 'vesicular budding' from Golgi membranes, and is essential for the retrograde Golgi-to-ER transport of dilysine-tagged proteins when COPE proteins increase in the absence of the other subunits this might exacerbate a movement of secreted proteins from the ribosome on the rough endoplasmic reticulum to the plasma membrane and so increase the secretion of proteins in EC.

CST3

CST3, also known as cystatin C is an inhibitor of several different cysteine proteinases and cathepsin B [12]. Cystatin C is a target for p53 where it is assumed that activation of this pathway results in increased pro-apoptotic functions in carcinogenesis [13]. An additional feature of relevance here is that CST3 is regulated by progesterone [14], which is well-known as a protective agent against EC [15].

C9orf142, and c14orf142

C9orf142, and c14orf142 are two proteins that appear to be involved in DNA repair mechanisms [16,17] and tRNA synthesis, respectively [18]. There is relatively little more known about the function of these proteins, except they are implicated in DNA repair and tRNA production through the EKC/KEOPS complex [18,19]. These data are not unexpected, since some of the cells in EC could be undergoing homeostatic regulation of DNA mutation or other forms of DNA damage and these genes are activated in an attempt to repair existing damage.

DKFZp313H139

DKFZp313H139 is a protein fragment that comes from a fusion region on Chromosome 6 that includes four protein members of the bromodomain family of proteins (BRD2, BRD3, BRD4 and BRDT), whose functions are unclear but could be related to the BET family of proteins that act as transcription regulators. BRD2 protein is known to regulate the acetylation of the retinoblastoma protein (a key anti-tumour protein). BRD3 is similar in structure to the RING3 protein, which is a SER/THR-kinase, but the actual function of BRD3 current remains unknown even though it has been identified in gastric carcinoma [20]. BRD4 is a protein that interrogates the DNA sequences in chromatin and binds acetylated histones [21]. By doing so, it aids the transmission of epigenetic memory across cell divisions and plays a critical role in the regulation of gene transcription [22]. It also acts as a regulator of p53/TP53-mediated transcription: following phosphorylation by CK2, it is recruited to p53/TP53 specific target promoters [23], to modulate the expression of the P53 protein, another tumour suppressor gene that is often mutated or has altered expression in EC [24]. BRDT is a known factor in ovarian cancer development where it promotes the expression of PLK1 and AURKC to stimulate cell cycle genes and activate the cell cycle [25]. It is also a regulator of EIFEBP1 in renal cell carcinoma where it acts a transcriptional regulator on its target gene's promoter [26]. It is likely that this peptide, identified in our study, could be key to EC carcinogenesis.

E7EPZ9-DECOY, Q8WZ42-12-DECOY and Q8WZ42-2-DECOY

The peptide fragments E7EPZ9-DECOY, Q8WZ42-12-DECOY and Q8WZ42-2-DECOY (which were used in the development of pharmaceuticals), were found to not have any known association with any biological function or cellular process and thus have no attendant literature source to draw upon. E7EPZ9-DECOY is now obsolete in the UniProt database (https://www.uniprot.org/), but now maps to TNXB (tenascin-X), which is an extracellular protein involved in the stabilisation of tissues and prevents cell migration [27]. It may also play a supporting role in the growth of some epithelial tumours [28], but that has yet to be confirmed. Q8WZ42-2-DECOY and Q8WZ42-12-DECOY are also obsolete in the

UniProt database and both sequences now map to titin (TTN) isoforms 2 and 12, respectively. Titin is the largest protein in the body (~3.8 million Da for isoform 2 and ~3.9 million Da for isoform 12) and is a key component in the assembly and normal functioning of striated muscles [29]. With regards to EC, it is likely that titin plays a role, although recent evidence of a direct effect disputes this statement [30]. Nevertheless, when fused to other RNA sequences such as the antisense sequence AS1, where the RNA for this molecule binds to the mRNA of titin, it has pro-oncogenic effects [31]. In this case, when TTN-AS1 is overexpressed, prognosis in a number of cancers, including those of the reproductive tract, but interestingly not for EC, are poor [31].

FAM169A

FAM169A, [Family With Sequence Similarity 169 Member A' or SLAP75 (Soluble Lamina-Associated Protein Of 75 KD)] is a nuclear envelope protein [32] that may have a role in intervertebral disc degeneration and apoptosis [33]. The precise role of this protein remains unknown, but mutant forms are associated with high BMI, a feature of EC patients [34].

GOLIM4

GOLIM4 (Golgi Integral Membrane Protein 4), also known as GPP130 is a type II Golgi-resident protein where its primary function is to process newly synthesised proteins and thus assists with the transport of cargo to the cell surface [35]. Although it does not directly interact with COPE, it performs similar functions to that protein, although its precise role even in hepatocarcinoma cells remains obscure [36].

GRN

GRN or progranulin/proepithelin, is a precursor protein that is secreted from the Golgi apparatus in neuronal tissue, but is targeted towards the lysosome where it has numerous functions including altering the activity of lysosomal enzymes [37,38] and lysosomal acidification [39]. This protein was identified in four different Reactome® networks and so it is not surprising that it may have additional roles, such as promoting epithelial cell proliferation by blocking TNF-mediated neutrophil activation preventing release of oxidants and proteases [40]. Moreover, it is known to modulate inflammation in neurons by preserving neuronal survival, axonal outgrowth and neuronal integrity [41]. We previously established that these functions are associated with APP, IGKV-I, IGKV-II, IGLV-III, CST3 and cathepsin D expression (**Supplemental Table S3**) and so can link several of the Reactome® networks through the expression of GRN.

HEXA

The enzyme hexosaminidase consists of two independent subunits HEXA (found to be overexpressed in EC) and HEXB, which is not. This enzyme is a member of the glycosyl hydrolase 20 family of proteins that catalyses the degradation of the ganglioside GM2, and other molecules containing terminal N-acetyl hexosamines [42]. HEXA is involved in the up-regulation of the cannabinoid receptor 2 (CNR2) during endometrial inflammation [43]; and we and others have reported CB2 expression to be altered in EC [44,45]. HEXA is also associated with GRN to ameliorate the lysosomal storage problems associated with Tay-Sachs disease suggesting that lysosomal function in EC is also affected [46].

HN1L

HN1L (Hematological and Neurological Expressed 1-Like Protein) is also known as JPT2 (Jupiter microtubule associated homolog 2). It is up-regulated in squamous cell carcinoma, adenocarcinoma, adenosquamous cell carcinoma, bronchioalveolar carcinoma of the lung [47], breast carcinoma [48] and is found in the uterus [49]. Furthermore, its native protein counterpart HN1 promotes tumour associated lymphangiogenesis and lymph node metastatic disease in patients with cervical cancer through activation of NF KappaB [50]. These data suggest this protein may a key role in EC pathogenesis.

IGKV-I, IGKV-II and IGLV-III

These 3 proteins (IGKV-I, IGKV-II and IGLV-III) are all immunoglobulin chains with the first two being part of the immunoglobulin kappa variable cluster, whilst the third is part of the lambda variable

cluster. These immunoglobulin light chains have no apparent functional differences, but mutations in these regions have been reported to be associated with certain types of lymphomas [51] and cancer cell lines [52].

J3QQ66-DECOY

The protein J3QQ66-DECOY has structural similarities to the murine ZFHX4 (Zinc finger homeobox 4) but cannot be found in any database as a single entity because ZFHX4 is a complex of four homeodomains and twenty-two zinc fingers [53], where they may act a key transcriptional regulators of downstream protein expression.

MYH8

MYH8 (Myosin Heavy Chain 8), is the major contractile protein found in striated muscle that associates with titin (see above) and may be dysfunctional in a number of conditions including Carney complex, which is a multiple neoplasia syndrome characterised by spotty skin pigmentation, cardiac and other myxomas, endocrine tumours, and psammomatous melanotic Schwannomas [54] due to defects in the PRKAR1A gene product [55], which was over-expressed in our EC patients (**Supplemental Table S1**) as was PRKAR2A an essential component of the protein kinase A complex [56].

PIGT

PIGT, also known as Phosphatidylinositol-Glycan Biosynthesis Class T Protein, is involved in glycosylphosphatidylinositol (GPI)-anchor biosynthesis. The GPI-anchor is a glycolipid found on many blood cells and serves to anchor proteins to the cell surface. The only existing evidence that PIGT is involved in cancer comes from a study whereby the PIGT subunit was silenced in breast cancer cell lines and resulted in the expression of membrane proteins many of which are identified in **Supplemental Table 2 and Tables 2 and 3** that have IgG domains and function in cell adhesion, protein-protein clustering, integrin activation, or antigen presentation [57].

PLCL1

PLCL1 [Phospholipase C Like 1 or Phospholipase C-Related Catalytically Inactive Protein 1 (PRIP-1)] is an inactive enzyme that shows no PLC activity to phosphatidylinositol 4,5-bisphosphate or phosphatidylinositol, but it regulates the turnover of GABA receptors and thus contributes to the maintenance of GABA-mediated synaptic inhibition [58]. Its aberrant expression could contribute to the genesis and progression of small cell lung carcinoma [59] where it acts as an inhibitor of PPP1C. In normal endometrial tissue, it is regulated directly by the actions of progesterone during stromal cell decidualisation, where it induces apoptosis and tissue breakdown via forkhead box protein O1 and the apoptosis activator BIM [60]. These data suggest that PLCL1 may be expressed in the stromal cell component of the EC tissue.

PMFBP1

PMFBP1 (Polyamine modulated factor 1 binding protein 1) has only one reported function of being intimately involved in normal spermatogenesis [61]) with abnormal expression of this protein producing sperm that lack a head (acephalic spermatozoa) because it acts as a scaffold protein that attaches the sperm head-tail connecting piece to the nuclear envelope, thus maintaining sperm head and tail integrity [62]. It is possible that it may be therefore involved in the normal cytoskeletal architecture of the endometrial cancer epithelial cell.

PPM1G

PPM1G, (Protein Phosphatase Magnesium-Dependent 1 Gamma) is a member of the PP2C family of Ser/Thr protein phosphatases that are known to be negative regulators of cell stress response pathways, especially when a cell is undergoing oxidative stress [63]. This phosphatase is found to be responsible for the dephosphorylation of pre-mRNA splicing factors, which is important for the formation of functional spliceosome [64] and *in-vitro* studies have demonstrated a role for this phosphatase in regulating cell cycle progression through degradation of the p27, a key cyclin-CDK inhibitor [65].

PPP1R2

PPP1R2, or Protein Phosphatase 1 Regulatory Inhibitor Subunit 2 is part of protein phosphatase-1 (PP1), which is one of the main Ser/Thr protein phosphatases, similar to PPM1G. PPP1R2 has an inhibitory role on PP1 and increased expression of these two proteins has been demonstrated in maintenance of central spindle stability during mitosis and increased chromosomal re-arrangements and may prevent cell abscission [66]. The result is stabilisation of cell numbers and tissue hyperplasia.

QARS, SARS and **WARS**

QARS and WARS are type 1 tRNA synthases, with QARS (Glutaminyl-tRNA Synthetase 1) adding glutamine to its cognate tRNA, whilst WARS (Tryptophanyl-tRNA Synthetase 1) adds tryptophan to its cognate tRNA and thus have important roles in protein synthesis [67]. SARS2 (Seryl-tRNA Synthetase 2, Mitochondrial) and MARS (Methionyl-tRNA Synthetase 1) were also overexpressed in this patient cohort (**Supplemental Table 1**), where the former adds serine to its cognate tRNA and is associated with AIDS progression [68]. By contrast, MARS has been shown to affect PPAR-alpha expression and acts as a growth inhibitor in the EC cell line, Ishikawa [69]. Whether changes in SARS2, QARS and WARS expression has any effect ~~on in~~on EC cell proliferation, is currently unknown, but based on the effect of MARS *in-vitro* and the over-expression in the current study, an effect is increasingly likely.

SCARB2

SCARB2 or Scavenger Receptor Class B Member 2, is also known as Lysosome Membrane Protein 2 or LIMP-2 (amongst others) is a type III glycoprotein primarily involved in limiting membranes of lysosomes and endosomes where it acts as a lysosomal receptor for glucosylceramidase [70] and is involved in cholesterol transport [71]. It is a ubiquitously expressed protein involved in the pathogenesis of HFMD (hand, foot, and mouth disease) caused by enterovirus-71 [72] and possibly by Coxsackie virus A16. It is possible that this protein is being used to transport cholesterol for ApoC1, which is also over-expressed in this patient cohort, where it might initiate wnt signalling pathways as it does in renal cancer [73]. Recent evidence also indicates that ApoC1 may be a good prognostic biomarker for cervical cancer [74].

SCPEP1

SCPEP1 (Serine Carboxypeptidase 1) is a retinoid-inducible serine carboxypeptidase that currently has no known function. It May be involved in vascular wall and kidney homeostasis by similarity to other proteins [75] and because it has only been found located in extracellular exosome.

SIAE

The enzyme Sialic Acid Acetylesterase (SIAE), which was also over-expressed in our patient cohort, removes 9- and 4-O-acetylation modifications from sialic acids [76] and especially from its parent molecule N-acetylneuraminic acid [77]. Acetylation and de-acetylation by SIAE have been implicated in colorectal carcinoma [78], creating the correct environment for coronavirus-SARS2 entry into cells [79] and is upregulated in preeclampsia and autoimmune diseases [80]. A key function of this enzyme is to regulate ganglioside-mediated apoptosis [81], and may link this to the function of HEXA (see above).

SLC25A24

The solute carrier protein SLC25A24 (Solute Carrier Family 25 Member 24) was also over-expressed in our cancer patients' samples. Its main function is to transport ATP-Mg^{2+} exchanging it for phosphate in the transport process across mitochondrial inner membranes [82]. ATP-Mg^{2+} is an important molecule in DNA synthesis and since a rapidly dividing cell increases its mitochondrial DNA synthesis, then it is not surprising that it would be increased in EC, because DNA damage in mitochondria results in increased apoptosis. It may do this by protecting cells against oxidative stress-induced cell death, probably by promoting the formation of calcium-phosphate precipitates in the mitochondrial matrix, and thereby buffering calcium levels in the mitochondrial matrix [83].

ZC3H4

ZC3H4 (Zinc Finger CCCH-Type Containing 4), can be found on chromosome 19 and is a member of a family of CCCH (C-x8-C-x5-C-x3-H type) zinc finger domain-containing proteins that coordinate zinc finger binding. They either act as RNA polymerase II-specific transcriptional regulators or function in proof-reading in post-transcriptional regulation, where they promote epithelial-mesenchymal transition and metastasis [84]. Over-expression is associated with obesity indices [85], due to increased body fat composition, a feature of women with a number of differing cancers, including EC [86]. It is likely therefore that ZC3H4 over-expression is linked to the slightly higher BMI identified in our patient cohort and the more aggressive nature of the type 2 EC that constituted the higher BMI group. It is also possible that APP, CAD, BRD2, CST3, GRN, PPM1G and ZC3H4 may contribute here, since these proteins have been implicated in adiposity and obesity in other situations [87-93].

References

1. Sosa, L.J.; Caceres, A.; Dupraz, S.; Oksdath, M.; Quiroga, S.; Lorenzo, A. The physiological role of the amyloid precursor protein as an adhesion molecule in the developing nervous system. *J Neurochem* **2017**, *143*, 11-29, doi:10.1111/jnc.14122.
2. Baumkötter, F.; Schmidt, N.; Vargas, C.; Schilling, S.; Weber, R.; Wagner, K.; Fiedler, S.; Klug, W.; Radzimanowski, J.; Nickolaus, S.; et al. Amyloid precursor protein dimerization and synaptogenic function depend on copper binding to the growth factor-like domain. *J Neurosci* **2014**, *34*, 11159-11172, doi:10.1523/jneurosci.0180-14.2014.
3. Higgs, M.R.; Reynolds, J.J.; Winczura, A.; Blackford, A.N.; Borel, V.; Miller, E.S.; Zlatanou, A.; Nieminuszczy, J.; Ryan, E.L.; Davies, N.J.; et al. BOD1L Is Required to Suppress Deleterious Resection of Stressed Replication Forks. *Mol Cell* **2015**, *59*, 462-477, doi:10.1016/j.molcel.2015.06.007.
4. Sigoillot, F.D.; Kotsis, D.H.; Serre, V.; Sigoillot, S.M.; Evans, D.R.; Guy, H.I. Nuclear localization and mitogen-activated protein kinase phosphorylation of the multifunctional protein CAD. *J Biol Chem* **2005**, *280*, 25611-25620, doi:10.1074/jbc.M504581200.
5. Staples, C.J.; Myers, K.N.; Beveridge, R.D.; Patil, A.A.; Howard, A.E.; Barone, G.; Lee, A.J.; Swanton, C.; Howell, M.; Maslen, S.; et al. Ccdc13 is a novel human centriolar satellite protein required for ciliogenesis and genome stability. *J Cell Sci* **2014**, *127*, 2910-2919, doi:10.1242/jcs.147785.
6. Blum, M.; Ott, T. Animal left-right asymmetry. *Curr Biol* **2018**, *28*, R301-r304, doi:10.1016/j.cub.2018.02.073.
7. Kaksonen, M.; Roux, A. Mechanisms of clathrin-mediated endocytosis. *Nat Rev Mol Cell Biol* **2018**, *19*, 313-326, doi:10.1038/nrm.2017.132.
8. Tsygankova, O.M.; Keen, J.H. A unique role for clathrin light chain A in cell spreading and migration. *J Cell Sci* **2019**, *132*, doi:10.1242/jcs.224030.
9. Konno, K.; Wakabayashi, Y.; Akashi-Takamura, S.; Ishii, T.; Kobayashi, M.; Takahashi, K.; Kusumoto, Y.; Saitoh, S.; Yoshizawa, Y.; Miyake, K. A molecule that is associated with Toll-like receptor 4 and regulates its cell surface expression. *Biochem Biophys Res Commun* **2006**, *339*, 1076-1082, doi:10.1016/j.bbrc.2005.11.123.
10. Lupi, L.A.; Cucielo, M.S.; Silveira, H.S.; Gaiotte, L.B.; Cesario, R.C.; Seiva, F.R.F.; de Almeida Chuffa, L.G. The role of Toll-like receptor 4 signaling pathway in ovarian, cervical, and endometrial cancers. *Life Sci* **2020**, *247*, 117435, doi:10.1016/j.lfs.2020.117435.
11. Garcia-Silva, M.R.; das Neves, R.F.; Cabrera-Cabrera, F.; Sanguinetti, J.; Medeiros, L.C.; Robello, C.; Naya, H.; Fernandez-Calero, T.; Souto-Padron, T.; de Souza, W.; et al. Extracellular vesicles shed by Trypanosoma cruzi are linked to small RNA pathways, life cycle regulation, and susceptibility to infection of mammalian cells. *Parasitol Res* **2014**, *113*, 285-304, doi:10.1007/s00436-013-3655-1.

12. Nagai, A.; Ryu, J.K.; Terashima, M.; Tanigawa, Y.; Wakabayashi, K.; McLarnon, J.G.; Kobayashi, S.; Masuda, J.; Kim, S.U. Neuronal cell death induced by cystatin C in vivo and in cultured human CNS neurons is inhibited with cathepsin B. *Brain Res* **2005**, *1066*, 120-128, doi:10.1016/j.brainres.2005.10.063.
13. Mori, J.; Tanikawa, C.; Funauchi, Y.; Lo, P.H.; Nakamura, Y.; Matsuda, K. Cystatin C as a p53-inducible apoptotic mediator that regulates cathepsin L activity. *Cancer Sci* **2016**, *107*, 298-306, doi:10.1111/cas.12881.
14. Song, G.; Spencer, T.E.; Bazer, F.W. Progesterone and interferon-tau regulate cystatin C in the endometrium. *Endocrinology* **2006**, *147*, 3478-3483, doi:10.1210/en.2006-0122.
15. Gambrell, R.D., Jr.; Bagnell, C.A.; Greenblatt, R.B. Role of estrogens and progesterone in the etiology and prevention of endometrial cancer: review. *Am J Obstet Gynecol* **1983**, *146*, 696-707, doi:10.1016/0002-9378(83)91014-1.
16. Craxton, A.; Somers, J.; Munnur, D.; Jukes-Jones, R.; Cain, K.; Malewicz, M. XLS (c9orf142) is a new component of mammalian DNA double-stranded break repair. *Cell Death Differ* **2015**, *22*, 890-897, doi:10.1038/cdd.2015.22.
17. Raschella, G.; Melino, G.; Malewicz, M. New factors in mammalian DNA repair-the chromatin connection. *Oncogene* **2017**, *36*, 4673-4681, doi:10.1038/onc.2017.60.
18. Drew, K.; Muller, C.L.; Bonneau, R.; Marcotte, E.M. Identifying direct contacts between protein complex subunits from their conditional dependence in proteomics datasets. *PLoS Comput Biol* **2017**, *13*, e1005625, doi:10.1371/journal.pcbi.1005625.
19. Wan, L.C.; Maisonneuve, P.; Szilard, R.K.; Lambert, J.P.; Ng, T.F.; Manczyk, N.; Huang, H.; Laister, R.; Caudy, A.A.; Gingras, A.C.; et al. Proteomic analysis of the human KEOPS complex identifies C14ORF142 as a core subunit homologous to yeast Gon7. *Nucleic Acids Res* **2017**, *45*, 805-817, doi:10.1093/nar/gkw1181.
20. Cho, J.; Ahn, S.; Son, D.S.; Kim, N.K.; Lee, K.W.; Kim, S.; Lee, J.; Park, S.H.; Park, J.O.; Kang, W.K.; et al. Bridging genomics and phenomics of gastric carcinoma. *Int J Cancer* **2019**, *145*, 2407-2417, doi:10.1002/ijc.32228.
21. Wang, R.; Li, Q.; Helfer, C.M.; Jiao, J.; You, J. Bromodomain protein Brd4 associated with acetylated chromatin is important for maintenance of higher-order chromatin structure. *J Biol Chem* **2012**, *287*, 10738-10752, doi:10.1074/jbc.M111.323493.
22. Patel, M.C.; Debrosse, M.; Smith, M.; Dey, A.; Huynh, W.; Sarai, N.; Heightman, T.D.; Tamura, T.; Ozato, K. BRD4 coordinates recruitment of pause release factor P-TEFb and the pausing complex NELF/DSIF to regulate transcription elongation of interferon-stimulated genes. *Mol Cell Biol* **2013**, *33*, 2497-2507, doi:10.1128/mcb.01180-12.
23. Wu, S.Y.; Lee, A.Y.; Lai, H.T.; Zhang, H.; Chiang, C.M. Phospho switch triggers Brd4 chromatin binding and activator recruitment for gene-specific targeting. *Mol Cell* **2013**, *49*, 843-857, doi:10.1016/j.molcel.2012.12.006.
24. Liu, Y.; Zhao, R.; Chi, S.; Zhang, W.; Xiao, C.; Zhou, X.; Zhao, Y.; Wang, H. UBE2C is upregulated by estrogen and promotes epithelial-mesenchymal transition via p53 in endometrial cancer. *Mol Cancer Res* **2020**, *18*, 204-215, doi:10.1158/1541-7786.Mcr-19-0561.
25. Chen, L.; Cai, S.; Wang, J.M.; Huai, Y.Y.; Lu, P.H.; Chu, Q. BRDT promotes ovarian cancer cell growth. *Cell Death Dis* **2020**, *11*, 1021, doi:10.1038/s41419-020-03225-y.
26. Wan, P.; Chen, Z.; Zhong, W.; Jiang, H.; Huang, Z.; Peng, D.; He, Q.; Chen, N. BRDT is a novel regulator of eIF4EBP1 in renal cell carcinoma. *Oncol Rep* **2020**, *44*, 2475-2486, doi:10.3892/or.2020.7796.
27. Chiquet-Ehrismann, R. Tenascins. *Int J Biochem Cell Biol* **2004**, *36*, 986-990, doi:10.1016/j.biocel.2003.12.002.
28. Egging, D.; van den Berkmortel, F.; Taylor, G.; Bristow, J.; Schalkwijk, J. Interactions of human tenascin-X domains with dermal extracellular matrix molecules. *Arch Dermatol Res* **2007**, *298*, 389-396, doi:10.1007/s00403-006-0706-9.

29. van der Loop, F.T.; van der Ven, P.F.; Furst, D.O.; Gautel, M.; van Eys, G.J.; Ramaekers, F.C. Integration of titin into the sarcomeres of cultured differentiating human skeletal muscle cells. *Eur J Cell Biol* **1996**, *69*, 301-307.
30. Liu, J.; Mei, J.; Li, S.; Wu, Z.; Zhang, Y. Establishment of a novel cell cycle-related prognostic signature predicting prognosis in patients with endometrial cancer. *Cancer Cell Int* **2020**, *20*, 329, doi:10.1186/s12935-020-01428-z.
31. Zheng, Q.X.; Wang, J.; Gu, X.Y.; Huang, C.H.; Chen, C.; Hong, M.; Chen, Z. TTN-AS1 as a potential diagnostic and prognostic biomarker for multiple cancers. *Biomed Pharmacother* **2021**, *135*, 111169, doi:10.1016/j.biopha.2020.111169.
32. Roux, K.J.; Kim, D.I.; Raida, M.; Burke, B. A promiscuous biotin ligase fusion protein identifies proximal and interacting proteins in mammalian cells. *J Cell Biol* **2012**, *196*, 801-810, doi:10.1083/jcb.201112098.
33. Li, Y.; Pan, D.; Liu, S.; Xing, X.; Zhou, H.; Zhang, B.; Zhang, D.; Li, B.; Li, G.; Tao, B.; et al. Identification of circ-FAM169A sponges miR-583 involved in the regulation of intervertebral disc degeneration. *J Orthop Translat* **2021**, *26*, 121-131, doi:10.1016/j.jot.2020.07.007.
34. Shaw, E.; Farris, M.; McNeil, J.; Friedenreich, C. Obesity and Endometrial Cancer. *Recent Results Cancer Res* **2016**, *208*, 107-136, doi:10.1007/978-3-319-42542-9_7.
35. Bachert, C.; Lee, T.H.; Linstedt, A.D. Lumenal endosomal and Golgi-retrieval determinants involved in pH-sensitive targeting of an early Golgi protein. *Mol Biol Cell* **2001**, *12*, 3152-3160, doi:10.1091/mbc.12.10.3152.
36. Duval, S.; Abu-Thuraia, A.; Elkholi, I.E.; Chen, R.; Seebun, D.; Mayne, J.; Cote, J.F.; Figeys, D.; Seidah, N.G. Shedding of cancer susceptibility candidate 4 by the convertases PC7/furin unravels a novel secretory protein implicated in cancer progression. *Cell Death Dis* **2020**, *11*, 665, doi:10.1038/s41419-020-02893-0.
37. Beel, S.; Moisse, M.; Damme, M.; De Muynck, L.; Robberecht, W.; Van Den Bosch, L.; Saftig, P.; Van Damme, P. Progranulin functions as a cathepsin D chaperone to stimulate axonal outgrowth in vivo. *Hum Mol Genet* **2017**, *26*, 2850-2863, doi:10.1093/hmg/ddx162.
38. Zhou, X.; Sun, L.; Bracko, O.; Choi, J.W.; Jia, Y.; Nana, A.L.; Brady, O.A.; Hernandez, J.C.C.; Nishimura, N.; Seeley, W.W.; et al. Impaired prosaposin lysosomal trafficking in frontotemporal lobar degeneration due to progranulin mutations. *Nat Commun* **2017**, *8*, 15277, doi:10.1038/ncomms15277.
39. Tanaka, Y.; Suzuki, G.; Matsuwaki, T.; Hosokawa, M.; Serrano, G.; Beach, T.G.; Yamanouchi, K.; Hasegawa, M.; Nishihara, M. Progranulin regulates lysosomal function and biogenesis through acidification of lysosomes. *Hum Mol Genet* **2017**, *26*, 969-988, doi:10.1093/hmg/ddx011.
40. Zhu, J.; Nathan, C.; Jin, W.; Sim, D.; Ashcroft, G.S.; Wahl, S.M.; Lacomis, L.; Erdjument-Bromage, H.; Tempst, P.; Wright, C.D.; et al. Conversion of proepithelin to epithelins: roles of SLPI and elastase in host defense and wound repair. *Cell* **2002**, *111*, 867-878, doi:10.1016/s0092-8674(02)01141-8.
41. Van Damme, P.; Van Hoecke, A.; Lambrechts, D.; Vanacker, P.; Bogaert, E.; van Swieten, J.; Carmeliet, P.; Van Den Bosch, L.; Robberecht, W. Progranulin functions as a neurotrophic factor to regulate neurite outgrowth and enhance neuronal survival. *J Cell Biol* **2008**, *181*, 37-41, doi:10.1083/jcb.200712039.
42. Cordeiro, P.; Hechtman, P.; Kaplan, F. The GM2 gangliosidoses databases: allelic variation at the HEXA, HEXB, and GM2A gene loci. *Genet Med* **2000**, *2*, 319-327, doi:10.1097/00125817-200011000-00003.
43. Iuvone, T.; De Filippis, D.; Di Spiezio Sardo, A.; D'Amico, A.; Simonetti, S.; Sparice, S.; Esposito, G.; Bifulco, G.; Insabato, L.; Nappi, C.; et al. Selective CB2 up-regulation in women affected by endometrial inflammation. *J Cell Mol Med* **2008**, *12*, 661-670, doi:10.1111/j.1582-4934.2007.00085.x.

44. Ayakannu, T.; Taylor, A.H.; Konje, J.C. Cannabinoid receptor expression in estrogen-dependent and estrogen-independent endometrial cancer. *J Recept Signal Transduct Res* **2018**, *38*, 385-392, doi:10.1080/10799893.2018.1531890.
45. Guida, M.; Ligresti, A.; De Filippis, D.; D'Amico, A.; Petrosino, S.; Cipriano, M.; Bifulco, G.; Simonetti, S.; Orlando, P.; Insabato, L.; et al. The levels of the endocannabinoid receptor CB2 and its ligand 2-arachidonoylglycerol are elevated in endometrial carcinoma. *Endocrinology* **2010**, *151*, 921-928, doi:10.1210/en.2009-0883.
46. Chen, Y.; Jian, J.; Hettinghouse, A.; Zhao, X.; Setchell, K.D.R.; Sun, Y.; Liu, C.J. Progranulin associates with hexosaminidase A and ameliorates GM2 ganglioside accumulation and lysosomal storage in Tay-Sachs disease. *J Mol Med (Berl)* **2018**, *96*, 1359-1373, doi:10.1007/s00109-018-1703-0.
47. Petroziello, J.; Yamane, A.; Westendorf, L.; Thompson, M.; McDonagh, C.; Cerveny, C.; Law, C.L.; Wahl, A.; Carter, P. Suppression subtractive hybridization and expression profiling identifies a unique set of genes overexpressed in non-small-cell lung cancer. *Oncogene* **2004**, *23*, 7734-7745, doi:10.1038/sj.onc.1207921.
48. Lo, Y.C.; Cormier, O.; Liu, T.; Nettles, K.W.; Katzenellenbogen, J.A.; Stearns, T.; Altman, R.B. Pocket similarity identifies selective estrogen receptor modulators as microtubule modulators at the taxane site. *Nat Commun* **2019**, *10*, 1033, doi:10.1038/s41467-019-08965-w.
49. Zhou, G.; Wang, J.; Zhang, Y.; Zhong, C.; Ni, J.; Wang, L.; Guo, J.; Zhang, K.; Yu, L.; Zhao, S. Cloning, expression and subcellular localization of HN1 and HN1L genes, as well as characterization of their orthologs, defining an evolutionarily conserved gene family. *Gene* **2004**, *331*, 115-123, doi:10.1016/j.gene.2004.02.025.
50. Chen, J.; Qiu, J.; Li, F.; Jiang, X.; Sun, X.; Zheng, L.; Zhang, W.; Li, H.; Wu, H.; Ouyang, Y.; et al. HN1 promotes tumor associated lymphangiogenesis and lymph node metastasis via NF-kappaB signaling activation in cervical carcinoma. *Biochem Biophys Res Commun* **2020**, *530*, 87-94, doi:10.1016/j.bbrc.2020.05.062.
51. Zhu, D.; Lossos, C.; Chapman-Fredricks, J.R.; Lossos, I.S. Biased immunoglobulin light chain use in the Chlamydophila psittaci negative ocular adnexal marginal zone lymphomas. *Am J Hematol* **2013**, *88*, 379-384, doi:10.1002/ajh.23416.
52. Hu, D.; Duan, Z.; Li, M.; Jiang, Y.; Liu, H.; Zheng, H.; Li, L.; Bode, A.M.; Dong, Z.; Cao, Y. Heterogeneity of aberrant immunoglobulin expression in cancer cells. *Cell Mol Immunol* **2011**, *8*, 479-485, doi:10.1038/cmi.2011.25.
53. Sakata, N.; Hemmi, K.; Kawaguchi, M.; Miura, Y.; Noguchi, S.; Ma, D.; Sasahara, M.; Kato, T.; Hori, M.; Tamaoki, T. The mouse ZFH-4 protein contains four homeodomains and twenty-two zinc fingers. *Biochem Biophys Res Commun* **2000**, *273*, 686-693, doi:10.1006/bbrc.2000.2990.
54. Stratakis, C.A.; Papageorgiou, T.; Premkumar, A.; Pack, S.; Kirschner, L.S.; Taymans, S.E.; Zhuang, Z.; Oelkers, W.H.; Carney, J.A. Ovarian lesions in Carney complex: clinical genetics and possible predisposition to malignancy. *J Clin Endocrinol Metab* **2000**, *85*, 4359-4366, doi:10.1210/jcem.85.11.6921.
55. Kamilaris, C.D.C.; Faucz, F.R.; Voutetakis, A.; Stratakis, C.A. Carney complex. *Exp Clin Endocrinol Diabetes* **2019**, *127*, 156-164, doi:10.1055/a-0753-4943.
56. Vincent-Dejean, C.; Cazabat, L.; Groussin, L.; Perlemoine, K.; Fumey, G.; Tissier, F.; Bertagna, X.; Bertherat, J. Identification of a clinically homogenous subgroup of benign cortisol-secreting adrenocortical tumors characterized by alterations of the protein kinase A (PKA) subunits and high PKA activity. *Eur J Endocrinol* **2008**, *158*, 829-839, doi:10.1530/EJE-07-0819.
57. Zhao, P.; Nairn, A.V.; Hester, S.; Moremen, K.W.; O'Regan, R.M.; Oprea, G.; Wells, L.; Pierce, M.; Abbott, K.L. Proteomic identification of glycosylphosphatidylinositol anchor-dependent

membrane proteins elevated in breast carcinoma. *J Biol Chem* **2012**, *287*, 25230-25240, doi:10.1074/jbc.M112.339465.
58. Fujii, M.; Kanematsu, T.; Ishibashi, H.; Fukami, K.; Takenawa, T.; Nakayama, K.I.; Moss, S.J.; Nabekura, J.; Hirata, M. Phospholipase C-related but catalytically inactive protein is required for insulin-induced cell surface expression of gamma-aminobutyric acid type A receptors. *J Biol Chem* **2010**, *285*, 4837-4846, doi:10.1074/jbc.M109.070045.
59. Kohno, T.; Otsuka, T.; Takano, H.; Yamamoto, T.; Hamaguchi, M.; Terada, M.; Yokota, J. Identification of a novel phospholipase C family gene at chromosome 2q33 that is homozygously deleted in human small cell lung carcinoma. *Hum Mol Genet* **1995**, *4*, 667-674, doi:10.1093/hmg/4.4.667.
60. Muter, J.; Brighton, P.J.; Lucas, E.S.; Lacey, L.; Shmygol, A.; Quenby, S.; Blanks, A.M.; Brosens, J.J. Progesterone-dependent induction of phospholipase C-related catalytically inactive protein 1 (PRIP-1) in decidualizing human endometrial stromal cells. *Endocrinology* **2016**, *157*, 2883-2893, doi:10.1210/en.2015-1914.
61. Sha, Y.W.; Wang, X.; Xu, X.; Ding, L.; Liu, W.S.; Li, P.; Su, Z.Y.; Chen, J.; Mei, L.B.; Zheng, L.K.; et al. Biallelic mutations in PMFBP1 cause acephalic spermatozoa. *Clin Genet* **2019**, *95*, 277-286, doi:10.1111/cge.13461.
62. Zhu, F.; Liu, C.; Wang, F.; Yang, X.; Zhang, J.; Wu, H.; Zhang, Z.; He, X.; Zhang, Z.; Zhou, P.; et al. Mutations in PMFBP1 Cause Acephalic Spermatozoa Syndrome. *Am J Hum Genet* **2018**, *103*, 188-199, doi:10.1016/j.ajhg.2018.06.010.
63. Pyo, J.; Ryu, J.; Kim, W.; Choi, J.S.; Jeong, J.W.; Kim, J.E. The protein phosphatase PPM1G destabilizes HIF-1α expression. *Int J Mol Sci* **2018**, *19*, doi:10.3390/ijms19082297.
64. Shkreta, L.; Chabot, B. The RNA splicing response to DNA damage. *Biomolecules* **2015**, *5*, 2935-2977, doi:10.3390/biom5042935.
65. Sun, C.; Wang, G.; Wrighton, K.H.; Lin, H.; Songyang, Z.; Feng, X.H.; Lin, X. Regulation of p27(Kip1) phosphorylation and G1 cell cycle progression by protein phosphatase PPM1G. *Am J Cancer Res* **2016**, *6*, 2207-2220.
66. Bresch, A.M.; Yerich, N.; Wang, R.; Sperry, A.O. The PP1 regulator PPP1R2 coordinately regulates AURKA and PP1 to control centrosome phosphorylation and maintain central spindle architecture. *BMC Mol Cell Biol* **2020**, *21*, 84, doi:10.1186/s12860-020-00327-5.
67. Yang, X.L.; Otero, F.J.; Ewalt, K.L.; Liu, J.; Swairjo, M.A.; Köhrer, C.; RajBhandary, U.L.; Skene, R.J.; McRee, D.E.; Schimmel, P. Two conformations of a crystalline human tRNA synthetase-tRNA complex: implications for protein synthesis. *Embo j* **2006**, *25*, 2919-2929, doi:10.1038/sj.emboj.7601154.
68. Hendrickson, S.L.; Lautenberger, J.A.; Chinn, L.W.; Malasky, M.; Sezgin, E.; Kingsley, L.A.; Goedert, J.J.; Kirk, G.D.; Gomperts, E.D.; Buchbinder, S.P.; et al. Genetic variants in nuclear-encoded mitochondrial genes influence AIDS progression. *PLoS One* **2010**, *5*, e12862, doi:10.1371/journal.pone.0012862.
69. Saidi, S.A.; Holland, C.M.; Charnock-Jones, D.S.; Smith, S.K. In vitro and in vivo effects of the PPAR-alpha agonists fenofibrate and retinoic acid in endometrial cancer. *Mol Cancer* **2006**, *5*, 13, doi:10.1186/1476-4598-5-13.
70. Reczek, D.; Schwake, M.; Schroder, J.; Hughes, H.; Blanz, J.; Jin, X.; Brondyk, W.; Van Patten, S.; Edmunds, T.; Saftig, P. LIMP-2 is a receptor for lysosomal mannose-6-phosphate-independent targeting of beta-glucocerebrosidase. *Cell* **2007**, *131*, 770-783, doi:10.1016/j.cell.2007.10.018.
71. Heybrock, S.; Kanerva, K.; Meng, Y.; Ing, C.; Liang, A.; Xiong, Z.J.; Weng, X.; Ah Kim, Y.; Collins, R.; Trimble, W.; et al. Lysosomal integral membrane protein-2 (LIMP-2/SCARB2) is involved in lysosomal cholesterol export. *Nat Commun* **2019**, *10*, 3521, doi:10.1038/s41467-019-11425-0.

72. Zhou, D.; Zhao, Y.; Kotecha, A.; Fry, E.E.; Kelly, J.T.; Wang, X.; Rao, Z.; Rowlands, D.J.; Ren, J.; Stuart, D.I. Unexpected mode of engagement between enterovirus 71 and its receptor SCARB2. *Nat Microbiol* **2019**, *4*, 414-419, doi:10.1038/s41564-018-0319-z.
73. Jiang, H.; Tang, J.Y.; Xue, D.; Chen, Y.M.; Wu, T.C.; Zhuang, Q.F.; He, X.Z. Apolipoprotein C1 stimulates the malignant process of renal cell carcinoma via the Wnt3a signaling. *Cancer Cell Int* **2021**, *21*, 41, doi:10.1186/s12935-020-01713-x.
74. Shi, X.; Wang, J.; Dai, S.; Qin, L.; Zhou, J.; Chen, Y. Apolipoprotein C1 (APOC1): A novel diagnostic and prognostic biomarker for cervical cancer. *Onco Targets Ther* **2020**, *13*, 12881-12891, doi:10.2147/OTT.S280690.
75. Chen, J.; Streb, J.W.; Maltby, K.M.; Kitchen, C.M.; Miano, J.M. Cloning of a novel retinoid-inducible serine carboxypeptidase from vascular smooth muscle cells. *J Biol Chem* **2001**, *276*, 34175-34181, doi:10.1074/jbc.M104162200.
76. Orizio, F.; Damiati, E.; Giacopuzzi, E.; Benaglia, G.; Pianta, S.; Schauer, R.; Schwartz-Albiez, R.; Borsani, G.; Bresciani, R.; Monti, E. Human sialic acid acetyl esterase: Towards a better understanding of a puzzling enzyme. *Glycobiology* **2015**, *25*, 992-1006, doi:10.1093/glycob/cwv034.
77. Rogers, G.N.; Herrler, G.; Paulson, J.C.; Klenk, H.D. Influenza C virus uses 9-O-acetyl-N-acetylneuraminic acid as a high affinity receptor determinant for attachment to cells. *J Biol Chem* **1986**, *261*, 5947-5951.
78. Shen, Y.; Kohla, G.; Lrhorfi, A.L.; Sipos, B.; Kalthoff, H.; Gerwig, G.J.; Kamerling, J.P.; Schauer, R.; Tiralongo, J. O-acetylation and de-O-acetylation of sialic acids in human colorectal carcinoma. *Eur J Biochem* **2004**, *271*, 281-290, doi:10.1046/j.1432-1033.2003.03927.x.
79. Kim, C.H. SARS-CoV-2 evolutionary adaptation toward host entry and recognition of receptor O-acetyl sialylation in virus-host interaction. *Int J Mol Sci* **2020**, *21*, doi:10.3390/ijms21124549.
80. Tsai, S.; Hardison, N.E.; James, A.H.; Motsinger-Reif, A.A.; Bischoff, S.R.; Thames, B.H.; Piedrahita, J.A. Transcriptional profiling of human placentas from pregnancies complicated by preeclampsia reveals disregulation of sialic acid acetylesterase and immune signalling pathways. *Placenta* **2011**, *32*, 175-182, doi:10.1016/j.placenta.2010.11.014.
81. Mandal, C.; Schwartz-Albiez, R.; Vlasak, R. Functions and biosynthesis of O-acetylated sialic acids. *Top Curr Chem* **2015**, *366*, 1-30, doi:10.1007/128_2011_310.
82. Harborne, S.P.; King, M.S.; Crichton, P.G.; Kunji, E.R. Calcium regulation of the human mitochondrial ATP-Mg/Pi carrier SLC25A24 uses a locking pin mechanism. *Sci Rep* **2017**, *7*, 45383, doi:10.1038/srep45383.
83. Traba, J.; Del Arco, A.; Duchen, M.R.; Szabadkai, G.; Satrústegui, J. SCaMC-1 promotes cancer cell survival by desensitizing mitochondrial permeability transition via ATP/ADP-mediated matrix Ca(2+) buffering. *Cell Death Differ* **2012**, *19*, 650-660, doi:10.1038/cdd.2011.139.
84. Jiang, R.; Gao, Q.; Chen, M.; Yu, T. Elk-1 transcriptionally regulates ZC3H4 expression to promote silica-induced epithelial-mesenchymal transition. *Lab Invest* **2020**, *100*, 959-973, doi:10.1038/s41374-020-0419-2.
85. Speliotes, E.K.; Willer, C.J.; Berndt, S.I.; Monda, K.L.; Thorleifsson, G.; Jackson, A.U.; Lango Allen, H.; Lindgren, C.M.; Luan, J.; Mägi, R.; et al. Association analyses of 249,796 individuals reveal 18 new loci associated with body mass index. *Nat Genet* **2010**, *42*, 937-948, doi:10.1038/ng.686.
86. Arthur, R.S.; Dannenberg, A.J.; Kim, M.; Rohan, T.E. The association of body fat composition with risk of breast, endometrial, ovarian and colorectal cancers among normal weight participants in the UK Biobank. *Br J Cancer* **2021**, doi:10.1038/s41416-020-01210-y.
87. Belkina, A.C.; Denis, G.V. BET domain co-regulators in obesity, inflammation and cancer. *Nat Rev Cancer* **2012**, *12*, 465-477, doi:10.1038/nrc3256.
88. Bjune, J.I.; Haugen, C.; Gudbrandsen, O.; Nordbø, O.P.; Nielsen, H.J.; Våge, V.; Njølstad, P.R.; Sagen, J.V.; Dankel, S.N.; Mellgren, G. IRX5 regulates adipocyte amyloid precursor protein

and mitochondrial respiration in obesity. *Int J Obes (Lond)* **2019**, *43*, 2151-2162, doi:10.1038/s41366-018-0275-y.
89. Cornelis, M.C.; Rimm, E.B.; Curhan, G.C.; Kraft, P.; Hunter, D.J.; Hu, F.B.; van Dam, R.M. Obesity susceptibility loci and uncontrolled eating, emotional eating and cognitive restraint behaviors in men and women. *Obesity (Silver Spring)* **2014**, *22*, E135-141, doi:10.1002/oby.20592.
90. Deng, Y.; Wang, Z.V.; Gordillo, R.; Zhu, Y.; Ali, A.; Zhang, C.; Wang, X.; Shao, M.; Zhang, Z.; Iyengar, P.; et al. Adipocyte Xbp1s overexpression drives uridine production and reduces obesity. *Mol Metab* **2018**, *11*, 1-17, doi:10.1016/j.molmet.2018.02.013.
91. Hooton, H.; Dubern, B.; Henegar, C.; Paternoster, L.; Nohr, E.A.; Alili, R.; Rousseau, F.; Pelloux, V.; Galan, P.; Hercberg, S.; et al. Association between CST3 rs2424577 polymorphism and corpulence related phenotypes during lifetime in populations of European ancestry. *Obes Facts* **2011**, *4*, 131-144, doi:10.1159/000327797.
92. Steinberg, G.R. Inflammation in obesity is the common link between defects in fatty acid metabolism and insulin resistance. *Cell Cycle* **2007**, *6*, 888-894, doi:10.4161/cc.6.8.4135.
93. Zanardini, R.; Benussi, L.; Fostinelli, S.; Saraceno, C.; Ciani, M.; Borroni, B.; Padovani, A.; Binetti, G.; Ghidoni, R. Serum C-peptide, visfatin, resistin, and ghrelin are altered in sporadic and GRN-associated frontotemporal lobar degeneration. *J Alzheimers Dis* **2018**, *61*, 1053-1060, doi:10.3233/jad-170747.
