# Supplementary material for: Identification of Potentially Novel Molecular Targets of Endometrial Cancer Using a Non-Biased Proteomic Approach
Source: Cancers (Basel). 2023 Sep 21;15(18):4665. doi: 10.3390/cancers15184665 (PMC10527058; doi:10.3390/cancers15184665)
Supplement: Supplementary file 1 [file cancers-15-04665-s001.zip › Supplemental Table S1.pdf]

A.H. Taylor, J.C. Konje and T. Ayakannu – "Identification of Potentially Novel Molecular Targets of Endometrial Cancer Using a Non-Biased Proteomic Approach"

Supplemental Table S1. The top 100 proteins differentially up-regulated in endometrial cancer relative to the level in atrophic endometrium.

The proteins presented in this table were identified by using proteome analysis of 4 endometrial cancer samples and 4 atrophic (normal) endometrial biopsies. After peptide selection and *in silico* analyses of peptide signals the data were analysed through the Reactome® pathway database (https://reactome.org/) and PantherGO classification system version 15.0 (released 14-02-2020) that contains 15702 protein families divided into 123989 functionally distinct protein subfamilies (http://pantherdb.org/webservices/go/overrep.jsp) for biological characterisation.

Gene Symbol†	Accession Number‡	Protein name	Fold Change	p-value*
LAMP2 [B]	P13473	Lysosome-associated membrane glycoprotein 2	29.0	0.056
HIST1H1B [B]	P16401	Histone H1.5	25.0	0.038
LRRFIP1 [B]	Q32MZ4	Leucine-rich repeat flightless-interacting protein 1	20.0	0.049
EPRS [B]	P07814	Bifunctional glutamate/proline--tRNA ligase	18.0	0.048
RPLP2 [B]	H0YDD8	60S acidic ribosomal protein P2 (Fragment)	16.0	0.048
RPL3 [B]	P39023	60S ribosomal protein L3	15.0	0.042
GAA [B]	P10253	Lysosomal alpha-glucosidase	15.0	0.042
RPL17 [B]	J3KRX5 (+2)	60S ribosomal protein L17 (Fragment)	14.0	0.003
MARCKSL1 [B]	P49006	MARCKS-related protein	12.0	0.051
EIF3C [B]	H3BRV0 (+1)	Eukaryotic translation initiation factor 3 subunit C	12.0	0.021
EIF3A [B]	Q14152	Eukaryotic translation initiation factor 3 subunit A	11.0	0.055
LUC7L [B]	A8MYV2 (+1)	LUC7-like (S. cerevisiae)	11.0	0.035
GSR [B]	P00390	Glutathione reductase, mitochondrial	11.0	0.042
DYNC1I2 [B]	Q13409-6	Isoform 2F of Cytoplasmic dynein 1 intermediate chain 2	10.0	0.047
MLEC [B]	Q14165	Malectin	10.0	0.029
HSD17B4 [B]	E7ER27	Peroxisomal multifunctional enzyme type 2	9.0	0.056
PAFAH1B1 [B]	P43034	Platelet-activating factor acetylhydrolase IB subunit alpha	9.0	0.042
FLNB [B]	E7EN95 (+1)	Filamin-B	8.4	0.056
ASPH [B]	Q12797	Aspartyl/asparaginyl beta-hydroxylase	8.0	0.038
HSPH1 [B]	Q92598-2	Isoform Beta of Heat shock protein 105 kDa	8.0	0.018
ERO1L [B]	Q96HE7	ERO1-like protein alpha	7.5	0.048
COPB2 [B]	B4DZI8 (+1)	Coatomer protein complex, subunit beta 2 (Beta prime), isoform CRA_β	7.5	0.026
RRBP1 [B]	Q9P2E9	Ribosome-binding protein 1	7.1	0.004
STMN1 [B]	P16949	Stathmin	7.0	0.054
SCP2 [B]	P22307-8	Isoform 8 of Non-specific lipid-transfer protein	7.0	0.044
S100A16 [B]	Q96FQ6	Protein S100-A16	7.0	0.044
YBX3 [B]	P16989 (+1)	Y-box-binding protein 3	7.0	0.014
RPL7 [B]	P18124	60S ribosomal protein L7	6.5	0.057
SRSF6 [B]	Q13247	Serine/arginine-rich splicing factor 6	6.5	0.032
SRSF4 [B]	Q08170	Serine/arginine-rich splicing factor 4	6.5	0.018
LUC7L2 [B]	Q9Y383	Putative RNA-binding protein Luc7-like 2	6.3	0.046
SELENBP1 [B]	Q13228	Selenium-binding protein 1	6.2	0.036

TGOLN2 [B]	F8WBK2 (+5)	Trans-Golgi network integral membrane protein 2	6.0	0.059
KHSRP [B]	Q92945	Far upstream element-binding protein 2	6.0	0.023
IGKV2D-28 [B]	P06309	Ig kappa chain V-II region GM607 (Fragment)	6.0	0.021
CTSH [B]	P09668	Pro-cathepsin H	6.0	0.021
RAD23B [B]	P54727	UV excision repair protein RAD23 homolog B	6.0	0.017
LAP3 [B]	P28838	Cytosol aminopeptidase	5.5	0.034
CD74 [B]	H0YBZ2 (+2)	HLA class II histocompatibility antigen gamma chain (Fragment)	5.0	0.054
CHMP5 [B]	Q9NZZ3-2	Isoform 2 of Charged multivesicular body protein 5	5.0	0.054
NUP62 [B]	M0QXN5 (+1)	Nuclear pore glycoprotein p62	5.0	0.054
SUMO2 [B]	P61956	Small ubiquitin-related modifier 2	5.0	0.054
SUMO3 [B]	A8MU27 (+2)	Small ubiquitin-related modifier 3	5.0	0.054
NUCB1 [B]	Q02818	Nucleobindin-1	5.0	0.053
PLS1 [B]	Q14651	Plastin-1	5.0	0.049
RNASET2 [B]	D6REQ6 (+2)	Ribonuclease T2	5.0	0.037
FUS [B]	P35637 (+1)	RNA-binding protein FUS	5.0	0.033
CMPK1 [B]	P30085	UMP-CMP kinase	5.0	0.033
C19orf10 [B]	Q969H8	UPF0556 protein C19orf10	5.0	0.033
HLA-B [B]	Q29940	HLA class I histocompatibility antigen, B-59 alpha chain	4.7	0.044
FLOT1 [B]	B0V109	Flotillin-1 (Fragment)	4.5	0.049
LAMP1 [B]	P11279	Lysosome-associated membrane glycoprotein 1	4.4	0.039
ILF3 [B]	Q12906	Interleukin enhancer-binding factor 3	4.3	0.058
MARCKS [B]	P29966	Myristoylated alanine-rich C-kinase substrate	4.2	0.050
CAPS [B]	Q13938	Calcyphosin	4.2	0.031
YBX1 [B]	P67809	Nuclease-sensitive element-binding protein 1	4.1	<0.001
SND1 [B]	Q7KZF4	Staphylococcal nuclease domain-containing protein 1	4.0	0.055
PRPF8 [B]	Q6P2Q9	Pre-mRNA-processing-splicing factor 8	4.0	0.054
USO1 [B]	F5GYR8	General vesicular transport factor p115	4.0	0.007
HNRNPAB [B]	D6RBZ0	Heterogeneous nuclear ribonucleoprotein A/B	3.8	0.019
ST13P4 [B]	Q8IZP2	Putative protein FAM10A4	3.7	0.058
HP1BP3 [B]	Q5SSJ5	Heterochromatin protein 1-binding protein 3	3.6	0.059
TM20nm [B]	A6NL28	Putative tropomyosin alpha-3 chain-like protein	3.5	0.020
RPL7A [B]	P62424	60S ribosomal protein L7a	3.4	0.059
KRT18 [B]	P05783	Keratin, type I cytoskeletal 18	3.4	0.033
EPB41L2 [B]	E9PK52	Band 4.1-like protein 2	3.3	0.057
SRSF2 [B]	J3KP15 (+1)	Serine/arginine-rich-splicing factor 2 (Fragment)	3.1	0.033
PKM [B]	P14618-2	Isoform M1 of Pyruvate kinase PKM	3.0	0.046
EPCAM [B]	B5MCA4	Epithelial cell adhesion molecule	3.0	0.052
SCP2 [B]	P22307	Non-specific lipid-transfer protein	3.0	0.044
NOP58 [B]	Q9Y2X3	Nucleolar protein 58	3.0	0.039
SSR1 [B]	P43307	Translocon-associated protein subunit alpha	3.0	0.039
PKM [B]	P14618	Pyruvate kinase PKM	3.0	0.059
HNRNPU [B]	Q00839	Heterogeneous nuclear ribonucleoprotein U	2.8	0.035
CAT [B]	P04040	Catalase	2.8	0.047
RPS25 [B]	P62851	40S ribosomal protein S25	2.8	0.054

DHX15 [B]	O43143	Putative pre-mRNA-splicing factor ATP-dependent RNA helicase DHX15	2.7	0.050
TPT1 [B]	E9PJF7	Translationally-controlled tumor protein (Fragment)	2.6	0.053
NME1 [B]	P15531-2	Isoform 2 of Nucleoside diphosphate kinase A	2.6	0.049
POTEJ [B]	P0CG39	POTE ankyrin domain family member J	2.6	0.052
WFDC2 [B]	Q14508	WAP four-disulfide core domain protein 2	2.5	0.061
HSP90AB2P [B]	Q58FF8	Putative heat shock protein HSP 90-beta 2	2.4	0.043
PSMC2 [B]	P35998	26S protease regulatory subunit 7	2.3	0.054
CKAP4 [B]	Q07065	Cytoskeleton-associated protein 4	2.3	0.044
APOE [B]	P02649	Apolipoprotein E	2.2	0.054
PGRMC2 [B]	O15173	Membrane-associated progesterone receptor component 2	2.1	0.054
HSPA4 [B]	P34932	Heat shock 70 kDa protein 4	2.1	0.030
HYOU1 [B]	Q9Y4L1	Hypoxia up-regulated protein 1	2.1	0.040
CANX [B]	B4DGP8 (+1)	Calnexin	2.1	0.023
SRSF1 [B]	J3KTL2 (+1)	Serine/arginine-rich-splicing factor 1	2.0	0.044
KRT8 [B]	P05787	Keratin, type II cytoskeletal 8	2.0	0.032
NCL [2]	P19338	Nucleolin	1.9	0.001
ERP29 [B]	P30040	Endoplasmic reticulum resident protein 29	1.9	0.049
HSPA5 [B]	P11021	78 kDa glucose-regulated protein	1.9	0.038
CALR [B]	P27797	Calreticulin	1.9	0.015
P4HB [B]	I3L0S0	Protein disulfide-isomerase (Fragment)	1.9	0.047
EIF2S1 [B]	P05198	Eukaryotic translation initiation factor 2 subunit 1	1.9	0.038
PRKCSH [2]	K7ELL7	Glucosidase 2 subunit beta	1.7	0.031
D6RGY2 [B]	SRP9	Signal recognition particle 9 kDa protein	1.7	0.015
K7ELL7 [B]	MPO	Isoform H7 of Myeloperoxidase	1.7	0.031

[†]Superscript after each gene symbol denotes increased protein expression in Type 2 EC (2); or both types of EC (B); (N.B. none of the 100 top up-regulated proteins were increased in Type 1 EC alone); [‡]Uniprot protein accession number; [*]p-value = unpaired Student one-tailed *t*-test as using parameters presented by Dalman *et al*. (2012).

Dalman, M.R., Deeter, A., Nimishakavi, G. *et al.* Fold change and p-value cutoffs significantly alter microarray interpretations. *BMC Bioinformatics* **13** (Suppl 2), S11 (2012). https://doi.org/10.1186/1471-2105-13-S2-S11).
