# Supplementary material for: Identification of Potentially Novel Molecular Targets of Endometrial Cancer Using a Non-Biased Proteomic Approach
Source: Cancers (Basel). 2023 Sep 21;15(18):4665. doi: 10.3390/cancers15184665 (PMC10527058; doi:10.3390/cancers15184665)
Supplement: Supplementary file 1 [file cancers-15-04665-s001.zip › Supplemental Table S2.pdf]

A.H. Taylor, J.C. Konje and T. Ayakannu – "Identification of Potentially Novel Molecular Targets of Endometrial Cancer Using a Non-Biased Proteomic Approach"

Supplemental Table S2. Proteins identified as already being associated with endometrial cancer.

Reactome® Network	Proteins identified in network	PubMed id
Autophagy	TUBAL3	30857319
Cell cycle	AKAP9; ATRX; CDH1; CDKN1B; DAXX; ITGB3; NUP62; POLE3; RAD50; RANBP2; SMARCA5; SYNE1; SYNE2; TP53BP1; TUBAL3	28448305; 31157162; 25286960; 21454826; 31384126; 26384307; 27092881; 25224212; 27979878; 30684972 33633465; 26330482; 31787999; 32059776; 30857319
Cell-cell communication	CADM1; CDH1; CTNNB1; CTNND1; DST; F11R; ITGB4; JUP; PSAP	29185275; 25286960; 26645990; 23874806; 30935077; 30675285; 30846786; 23874806; 23874806
Cellular responses to external stimuli	CDH1; CDKN1B; HIST1H1A; HIST1H1D; HMGA1; HSPH1; MT2A; NUP62; PSMC2; RAD50; RANBP2; RPL10; RPL13A; RPL27A; RPL28; RPL29; RPLP1; RPS24; STAT3; TUBAL3	25286960; 21454826; 33177874; 33177874; 27001612; 32652986; 26485755; 27092881; 29130934; 27979878; 30684972; 21226949; 32807831; 20015385; 30888986; 26892688; 32547595; 19101990; 28114390; 30857319
Chromatin organisation	ACTL6A; CARM1; CHD3; CHD4; HDAC2; KMT2D; SMAP; SMARCC2; TAF15	31028182; 22876322; 29888111; 29888111; 22112317; 31864301; 30104481; 31028182; 25733820
Circadian clock	CARM1	22876322
Developmental Biology	ADAM10; ALCAM; CARM1; COL2A1; COL3A1; COL4A2; COL5A1; CTNNA2; CTNNB1; DEK; DSG2; HDAC2; ITGA2; ITGB3; JUP; KMT2D; LAMA1; MMP9; MYH14; PABPC1; PML; PRKAR2A; PRNP; PSMC2; PTPRC; RNPS1; RPL10; RPL13A; RPL27A; RPL28; RPL29; RPLP1; RPS24; STAT3; TUBAL3	25781554; 29682175; 22876322; 31268882; 19065659; 26849234; 32641130; 27409342; 26645990; 25733820; 30846786; 22112317; 25733820; 26384307; 23874806; 31864301; 24832084; 28114390; 33217970; 31607220; 28969912; 31850214; 32560489; 29130934; 25733820; 31355251; 21226949; 32807831; 20015385; 30888986; 26892688; 32547595; 19101990; 28114390; 30857319
Digestion and absorption	There are no proteins to report in this category.	There are no proteins to report in this category.
Disease	ABI1; ADAM10; AHCYL1; AKAP9; ATRX; B4GALT1; BRD4; CD14; CDH1; CDKN1B; CHD3; CHD4; CTNNB1; CTNND1; CYFIP2; DAXX; DNAJC3; ESR1; GBE1; GFPT1; GLB1; HDAC2; HLA-A; HMGA1;	25733820; 25781554; 26865667; 28448305; 31157162 31807118; 29941483; 25880896; 25286960; 21454826; 29888111; 29888111; 26645990; 23874806; 30935077; 31384126; 31853186; 23593326; 33424936; 29464034; 32894095; 22112317; 28358435; 27001612;

	IARS2; ITGB3; MAOA; MCCC2; MUC16; NUP62; PAPSS1; PARP1; PML; PRKAR1A; PRKAR2A; PSAP; PSMC2; RANBP2; RPIA; RPL10; RPL13A; RPL27A; RPL28; RPL29; RPLP1; RPS24; SLC16A1; SLC2A1; STAT3; TAF15; TCEA1; TUBAL3	25733820; 26384307; 25733820; 27391266; 31864301; 27092881; 22380844; 23762867; 28969912; 22461635; 31850214; 23874806; 29130934; 30684972; 30177747; 21226949; 32807831; 20015385; 30888986; 26892688; 32547595; 19101990; 29665298; 31720081; 28114390; 25733820; 32877461; 30857319
DNA repair	ACTL6A; PARP1; PGM3; POLE3; RAD23B; RAD50; SMARCA5; SMARCC2; SUMO2; SUMO3; TCEA1; TP53BP1; TRIM25; XPC	31028182; 23762867; 508567; 25224212; 22544315; 27979878; 33633465; 31028182; 25733820; 25733820; 32877461; 32059776; 30586414; 21750170
DNA replication	CDH1; ITGB3; MCM2; POLE3; PSMC2	25286960; 26384307; 31268882; 25224212; 29130934
Extracellular matrix organisation	ADAM10; CDH1; COL16A1; COL26A1; COL2A1; COL3A1; COL4A2; COL5A1; DST; F11R; FBN2; ITGA2; ITGAM; ITGB3; ITGB4; LAMA1; LTBP1; MME; MMP9; P4HA1; P4HA2; SPP1	25781554; 25286960; 25733820; 32315343; 31268882; 19065659; 26849234; 32641130; 30935077; 30675285; 25733820; 25733820; 33292231; 26384307; 30846786; 24832084; 23874806; 24623538; 28114390; 31036064; 30857319; 32679719
Gene expression (transcription)	ACTL6A; CARM1; CCDC47; CDH1; CDKN1B; CHD3; CHD4; CTNNB1; DAXX; DEK; ESR1; HDAC2; KMT2D; LEO1; NUP62; PARP1; PML; PSMC2; RAD50; RANBP2; RNPS1; SMARCA5; SMARCC2; SNRPB; SPP1; SRRM1; SRSF4; SRSF5; TAF15; TCEA1	31028182; 22876322; 22403726; 25286960; 21454826; 29888111; 29888111; 26645990; 31384126; 25733820; 23593326; 22112317; 31864301; 23755103; 27092881; 23762867; 28969912; 29130934; 27979878; 30684972; 31355251; 33633465; 31028182; 32983981; 32679719; 31911863; 31355251; 33163258; 25733820; 32877461
Haemostasis	CCDC47; CD63; CD74; CEACAM5; CLU; CYFIP2; DAXX; F11R; HDAC2; ITGA2; ITGAM; ITGB3; KIF15; PLAUR; PRKAR1A; PRKAR2A; PSAP; SLC16A1; SMAP; TUBAL3	22403726; 32555395; 33159014; 32899298; 31623106; 30935077; 31384126; 30675285; 22112317; 25733820; 33292231; 26384307; 29693365; 12130664; 22461635; 31850214; 23874806; 29665298; 30104481; 30857319
Immune system	ABI1; ACLY; ADAM10; AHCYL1; ALCAM; ATAD3B; B4GALT1; C1QC; CCDC47; CD14; CD63; CD74; CDH1; CDKN1B; CLU; COL2A1; COL3A1; CTNNB1; CTSH; CYFIP2; DNAJC3; DNAJC5; ERAP1; FCGR3B; FECH; GGH; GLB1;	25733820; 23454913; 25781554; 26865667; 29682175; 33177874; 31807118; 22403726; 22403726; 25880896; 32555395; 33159014; 25286960; 21454826; 31623106; 31268882; 19065659; 26645990; 22040021; 30935077; 31853186; 33338880; 31921832; 32339157; 31783595; 31105744; 32894095;

	GSTK1; HLA-A; HLA-DRB1; HUWE1; IFI30; IFITM1; IGF2R; ITGAM; JUP; KIAA1324; KIF15; LGMN; LTBR; MAOA; MME; MMP9; MT2A; MUC16; NUP62; OLFM4; PLAUR; PML; PSAP; PSMC2; PTPRC; RANBP2; S100A12; S100P; SMAP; SNAP23; STAT3; TRIM2; TRIM25; TRIP12; TUBAL3; TWF1	21458040; 28358435; 33159014; 31787999; 30203794; 27124937; 29297146; 33292231; 23874806; 32774497; 29693365; 16289307; 20554747; 25733820; 24623538; 28114390; 26485755; 31864301; 27092881; 24495253; 12130664; 28969912; 23874806; 29130934; 25733820; 30684972; 32339157; 24966918; 30104481; 28938593; 28114390; 25733820; 30586414; 33198194; 30857319; 24595016
Metabolism	ACLY; AHCYL1; AKR1C3; ALDH18A1; APOC2; B4GALT1; BCAT2; CARM1; CMPK1; CSE1L; CYB5A; CYC1; EPRS; FBP1; FECH; FLOT1; GBE1; GGCT; GLB1; GMPR; GSTK1; HGD; HPGD; IGF2R; LGMN; LTBP1; MAOA; MCCC2; NQO1; NUP62; OAT; PAPSS1; PLCB1; PRKAR1A; PRKAR2A; PSAP; PSMC2; RANBP2; RBKS; RNPS1; RPIA; RPL10; RPL13A; RPL27A; RPL28; RPL29; RPLP1; RPS24; SDHB; SLC16A1; SLC2A1; SPP1; SUMO2; TCEA1; TWF1	23454913; 26865667; 33352741; 32894095; 21489304; 31807118; 31949641; 22876322; 26716509; 31134156; 26328264; 32983981; 31788113; 29190956; 31783595; 26865667; 33424936; 31947591; 32894095; 23843231; 21458040; 28167798; 31692912; 29297146; 16289307; 23874806; 25733820; 27391266; 28748640; 27092881; 27181389; 22380844; 30813939; 22461635; 31850214; 23874806; 29130934; 30684972; 27219024; 31355251; 30177747; 21226949; 32807831; 20015385; 30888986; 26892688; 32547595; 19101990; 25376524; 29665298; 31720081; 32679719; 25733820; 32877461; 24595016
Metabolism of protein	ACTL6A; ADAM10; ALPL; DAXX; EIF5; ESR1; FCGR3B; FECH; GLB1; KIAA1324; MME; MUC16; PARP1; PGM3; PLAUR; PRNP; PSAP; RAB21; RANBP2; RPL10; RPL13A; RPL27A; RPS24; SPP1; STAT3; SYNE1; TES; TUBAL3; UBA2	31028182; 25781554; 29659871; 31384126; 32726992; 23593326; 32339157; 31783595; 32894095; 32774497; 24623538; 31864301; 23762867; 508567; 12130664; 32560489; 23874806; 19887623; 30684972; 21226949; 32807831; 20015385; 19101990; 32679719; 28114390; 26330482; 24929083; 30857319; 22923510
Metabolism of RNA	ATRX; EPRS; ERMP1; NUP62; PABPC1; PSMC2; RANBP2; RBMX; RNPS1; RPL10; RPL13A; RPL27A; RPL28; RPL29; RPLP1; RPS24; SET; SNRPB; SNRPD2; SRRM1; SRSF4; SRSF5; UPF1	31157162; 31788113; 29523216; 27092881; 31607220; 29130934; 30684972; 24170611; 31355251; 21226949; 32807831; 20015385; 30888986; 26892688;32547595; 19101990; 26351531; 32983981; 32899298; 31911863; 31355251; 33163258; 32210576

Muscle contraction	AHCYL1; AKAP9; ASPH; CALD1; LMOD1; MYH6; SORBS1; TTN	26865667; 28448305; 31934181; 20579378; 33312770; 33217970; 19583808; 31864301
Neuronal system	DNAJC5; FLOT1; MAOA; MYO6; NPTN; PLCB1; PRKAR1A; PRKAR2A; SORBS1; TUBAL3	33338880; 26865667; 25733820; 32471032; 27391266; 30813939; 22461635; 31850214; 19583808; 30857319
Organelle biogenesis and maintenance	AKAP9; CARM1; CYC1; IMMT; SMAP; TUBAL3	28448305; 22876322; 32983981; 32983981; 30104481; 30857319
Programmed cell death	CD14; CDH1; CTNNB1; DIABLO; DSG2; FLOT1; HIST1H1A; HIST1H1D; PSMC2; STAT3	25880896; 25286960; 26645990; 30887763; 30846786; 26865667; 33177874; 33177874; 29130934; 28114390
Protein localisation	CYB5A; CYC1; FIS1; GSTK1; PRNP	26328264; 32983981; 30018222; 21458040; 32560489
Reproduction	B4GALT1; RAD50; SMC1A; SYNE1; SYNE2	31807118; 27979878; 30886832; 26330482; 31787999
Signal transduction	ABI1; ADAM10; AHCYL1; AKAP9; AKR1C3; APOC2; B4GALT1; CARM1; CCDC47; CDH1; CDKN1B; CHD3; CHD4; COL2A1; COL3A1; COL4A2; COL5A1; CTNNB1; CTNND1; CYFIP2; ESR1; F11R; GAS1; GLB1; GSTK1; HDAC2; HSPH1; IARS2; ITGA2; ITGB3; JUP; KMT2D; LAMA1; LEO1; MDK; MMP9; MYH14; PARP1; PLCB1; PML; PRKAR1A; PRKAR2A; PSAP; PSMC2; RANBP2; SMC1A; SOX6; SPP1; STAT3; TCEA1; TFF3; TUBAL3	25733820; 25781554; 26865667; 28448305; 33352741; 21489304; 31807118; 22876322; 22403726; 25286960; 21454826; 29888111; 29888111; 31268882; 19065659; 26849234; 32641130; 26645990; 23874806; 30935077; 23593326; 30675285; 33053339; 32894095; 21458040; 22112317; 22403726; 25733820; 25733820; 26384307; 23874806; 31864301; 24832084; 23755103; 23874806; 28114390; 33217970; 23762867; 30813939; 28969912; 22461635; 31850214; 23874806; 29130934; 30684972; 30886832; 21976109; 32679719; 28114390; 32877461; 30550483; 30857319
Transport of small molecules	AHCYL1; ANO1; APOC2; ASPH; IGF2R; ITGA2; PRKAR1A; PRKAR2A; PSMC2; RANBP2; SLC16A1; SLC2A1; TFRC; UBA2	26865667; 30651795; 21489304; 31934181; 29297146; 25733820; 22461635; 31850214; 29130934; 30684972; 29665298; 31720081; 26308378; 22923510
Vesicle mediated transport	ALPP; ANO1; COL3A1; COL4A2; HIP1; HSPH1; IGF2R; KIF15; MYO6; NQO1; RAB21; RABGAP1; SMAP; SNAP23; TFRC; TUBAL3; TWF1	7829680; 30651795; 19065659; 26849234; 25733820; 22403726; 29297146; 29693365; 32471032; 28748640; 19887623; 29464034; 30104481; 28938593; 26308378; 30857319; 24595016
Found by GO pathway analysis but missing from	ABRACL; AGR3; ANKS1B; ARFIP1; BCLAF1; BRD3; CAPRIN1; CD276; CD82; CELF1; CIRBP; CMRK1;	30087246; 30140383; 29622464; 25733820; 18450952; 29941483; 31911863; 27391266; 19294001; 26606540; 33488112; 26716509;

Reactome® network analysis	COX7A2; CTSH; CYB5A; DNAH14; DSG2; EIF3J; FAM83G; FARP1; HDGFRP2; IGFBP2; LRBA; LSP1; LUC7L; MACF1; MAP1B; MTDH; NAV3; NEFM; NOL3; PAWR; PDS5B; PDZD2; PKHD1L1; PLXNB2; PNN; PODXL; RALGAPA1; REXO1; RRBP1; S100A16; SART3; SCARA3; SLC9A3R1; SLK; TACSTD2; TCEAL2; TCEAL4; TMEM165; TNFAIP2; TOP1; TTC1; YBX3; ZC3H13; ZRANB2	21458040; 22040021; 26328264; 29464034; 30846786; 31887549; 28078827; 20015385; 24692842; 26045339; 25733820; 21965274; 22403726; 30935077; 25733820; 21687633; 31787999; 28088687; 26042464; 28008141; 25733820; 31338870; 29194195; 32654393; 19784388; 32615943; 29464034; 29079795 30684972; 22559235; 11092984; 26559525; 32120995; 20015385; 21892093; 30655845; 29079795; 25733820; 26716509; 25733820; 32547595; 33602962; 23755103; 31256208
