# Supplementary material for: Identification of Potentially Novel Molecular Targets of Endometrial Cancer Using a Non-Biased Proteomic Approach
Source: Cancers (Basel). 2023 Sep 21;15(18):4665. doi: 10.3390/cancers15184665 (PMC10527058; doi:10.3390/cancers15184665)
Supplement: Supplementary file 1 [file cancers-15-04665-s001.zip › Supplemental Table S3.pdf]

A.H. Taylor, J.C. Konje and T. Ayakannu – "Identification of Potentially Novel Molecular Targets of Endometrial Cancer Using a Non-Biased Proteomic Approach"

Supplemental Table S3. Proteins not previously associated with endometrial cancer **BUT** known to be associated with other forms of cancer.

Reactome® Network	Proteins identified in network	EC Type*	PubMed id
Autophagy	ABCC6; DYNC1I2; LAMP2;<br>PCNT; UBA52	2; B; B;<br>2; B	31565477; 31516392; 27627761;<br>28426876; 27270322
Cell cycle	ARPP19; CAST; CETN2;<br>DCTN1; DKC1; DYNC1I2;<br>ENSA; NHP2; NUP205;<br>PBDC1; PCNT; PPP1R12A;<br>PSMB7; PSMD10; PSMD3;<br>PSMD6; PSMD8; RAB2A;<br>RCC1; RCC2; RPS27;<br>TMPO; UBA52; USO1	2; B; B;<br>B; 2; B;<br>B; 2; 2;<br>1; 2; 2;<br>B; 2; B;<br>2; 2; 1;<br>2; 2; B;<br>B; B; B	28361685; 11895905; 30375513;<br>33198314; 31857720; 31516392;<br>31348270; 28289249; 32467671;<br>25428507; 28426876; 26113782;<br>24758163; 36028821; 33193699;<br>26291246; 24889965; 26379393;<br>30280037; 28869598; 28923911;<br>33371267; 27270322; 28969018
Cell-cell communication	LAMA3	B	32555663
Cellular responses to external stimuli	ATP6V1A; DCTN1; DYNC1I2;<br>EIF2S2; EIF2S3; FASN;<br>HSBP1; KIAA1967; MT1X;<br>NUP205; PSMA3; PSMA6;<br>PSMB7; PSMD10; PSMD3;<br>PSMD6; PSMD8; RPL14;<br>RPL15; RPL17; RPL18A;<br>RPL19; RPL23A; RPL24;<br>RPL3; RPL32; RPL34;<br>RPL35; RPL35A; RPL36;<br>RPS11; RPS13; RPS21;<br>RPS23; RPS27; RPS28;<br>TXNRD1; UBA52	B; B; B;<br>B; B; 2;<br>B; B; 2;<br>2; B; B;<br>B; 2; B;<br>2; 2; 1;<br>1; B; B;<br>B: B; 1;<br>B; B; 1;<br>1; B; B;<br>B; B; B;<br>B; B; B;<br>1; B	28592880; 33198314; 31516392;<br>17989776; 29320577; 25947066;<br>28686225; 24206917; 25763113;<br>32467671; 29304754; 31086659;<br>24758163; 36028821; 33193699;<br>26291246; 24889965; 28264936;<br>26892688; 26597576; 30678426;<br>24950402; 28147343; 30650663;<br>29530001; 25864709; 29530001;<br>29530001; 29530001; 32640226;<br>32395510; 29530001; 27233751;<br>30061686; 28923911; 31350888;<br>20420945; 27270322
Chromatin organisation	HIST1H2AG; KDM5B	B; 2	25003966; 27480251
Circadian clock	UBA52; USP9X	B; 2	27270322; 28103826
Developmental Biology	ADIRF; APOC1; COL5A3;<br>DPPA4; EIF4G1; EVPL;<br>FARP2; FLNB; KRT16;<br>KRT6B; KRT77; MYL12B;<br>NCSTN; PSMA3; PSMA6;<br>PSMB7; PSMD10; PSMD3;<br>PSMD6; PSMD8; RPL14;<br>RPL15; RPL17; RPL18A;<br>RPL19; RPL23A; RPL24;<br>RPL3; RPL32; RPL34;<br>RPL35; RPL35A; RPL36;<br>RPS11; RPS13; RPS21;<br>RPS23; RPS27; RPS28;<br>SDC2; SPTBN2; SUMF2;	2; 1; 2;<br>2; B; 2;<br>2; B; B;<br>B; 2; B;<br>1; B; B;<br>B; 2; B;<br>2; 2; 1;<br>1; B; B;<br>B: B; 1;<br>B; B; 1;<br>1; B; B;<br>B; B; B;<br>B; B; B;<br>B; 2; B;	32596316; 30732558; 27597234;<br>23963736; 18427574; 29587367;<br>25285016; 30764547; 32083078;<br>25997063; 22278369; 25029196;<br>25887147; 29304754; 31086659;<br>24758163; 36028821; 33193699;<br>26291246; 24889965; 28264936;<br>26892688; 26597576; 30678426;<br>25300887; 28147343; 30650663;<br>30105457; 25864709; 29530001;<br>29530001; 28923911; 32640226;<br>26892688; 29530001; 27233751;<br>30061686; 28923911; 27741504;<br>26293675; 28793054; 3229626;

	THRAP3; TSC22D1; UBA52; USP9X	2; 2; B; 2	29112714; 23442798; 27270322; 28103826
Digestion and absorption	There are no proteins to report in this category.		
Disease	ABCC6; AP1G1; ATP1B3; CAST; CHMP5; CYFIP1; DYNC1I2; FASN; FLNB; GAA; GALE; GGT1; GOLGB1; HIST1H2AG; LRRFIP1; NCSTN; NUP205; PABPN1; PCCB; PSMA3; PSMA6; PSMB7; PSMD10; PSMD3; PSMD6; PSMD8; RCC1; RHOG; RPL14; RPL15; RPL17; RPL18A; RPL19; RPL23A; RPL24; RPL3; RPL32; RPL34; RPL35; RPL35A; RPL36; RPS11; RPS13; RPS21; RPS23; RPS27; RPS28; SDC1; SDC2; SGSH; SH3GL1; SLC25A4; TXNRD1; UBA52	2; B; 1; B; B; B; B; 2; B; B; B; 1; 2; B; B; 1; 2; 2; B; B; B; B; 2; B; 2; 2; 2; 2; 1; 1; B; B; B; B; B; B; B; 1; 1; B; B; B; B; B; B; B; B; 1; B; 2; B; B; 1; B	31565477; 31002129; 24236063; 11895905; 30678426; 29572294; 31516392; 25947066; 31665330; 8660303; 31272438; 27381639; 20685852; 25003966; 26224133; 25887147; 32467671; 30787966; 26627478; 29304754; 31086659; 24758163; 36028821; 33193699; 26291246; 24889965; 30280037; 28202690; 28264936; 26892688; 26597576; 30678426; 24950402; 28147343; 30650663; 29530001; 25864709; 29530001; 29530001; 29530001; 32640226; 26892688; 29530001; 27233751; 30061686; 28923911; 27741504; 29340066; 26293675; 31746421; 30246154; 26091520; 20420945; 27270322
DNA repair	CETN2; DDB1; RAD23A; UBA52; USP7	B; B; B; B; 2	30375513; 29594129; 29633253; 27270322; 32850836
DNA replication	PSMA3; PSMA6; PSMB7; PSMD10; PSMD3; PSMD6; PSMD8; UBA52	B; B; B; 2; B; 2; 2; B	29304754; 31086659; 24758163; 36028821; 33193699; 26291246; 24889965; 27270322
Extracellular matrix organisation	CAST; CEACAM1; COL21A1; COL5A3; EMILIN2; ITGB2; LAMA3; NCSTN; SDC1; SDC2; ZNF207	B; B; 2; 2; B; B; 2; 1; 1; B; 2	11895905; 24931667; 27390605; 27597234; 30544909; 32226771; 32555663; 25887147; 29340066; 26293675; 33563334
Gene expression (transcription)	APOC1; CAST; CNOT2; CNOT3; COX5B; DDX21; DNAJB11; G6PD; KDM5B; NDRG1; NUP205; PABPN1; PBDC1; PIP4K2A; PRKRA; PSMA3; PSMA6; PSMB7; PSMD10; PSMD3; PSMD6; PSMD8; RBM14; SRSF6; TAOK2; TP53I3; TXNRD1; UBA52; USP7; USP9X	1; B; 2; 2; B; 2; B; B; 2; B; 2; 2; 1; 1; B; B; B; B; 2; B; 2; 2; B; B; 2; 2; 1; B; 2; 2	30732558; 11895905; 30108113; 30650663; 26869356; 33328538; 21423607; 27861141; 27480251; 29898756; 32467671; 30787966; 25428507; 28637795; 33406242; 29304754; 31086659; 24758163; 36028821; 33193699; 26291246; 24889965; 28786813; 32901876; 31088413; 31223287; 20420945; 27270322; 32850836; 28103826
Haemostasis	ATP1B3; ATP2B1; CD58; CEACAM1; CYB5R1; EMILIN2; FLNB; GYPA; ITGB2; LAMP2; MAGED2; MMRN1; OLA1; PYCR1; RHOG; SDC1; SDC2; TMSB4X	1; B; 2; B; 2; B; B; 1; B; B; 2; 2; 2; 2; 2; 1; B; 2	24236063; 29285225; 22403726; 24931667; 27120783; 30544909; 30764547; 26716904; 32226771; 27627761; 32547595; 23536776; 26863455; 29805774; 28202690; 29340066; 26293675; 31781249
Immune system	ACTR1B; AGL; AP1G1; APEH; ASAH1; ATP6V1A; BPIFA1; BST2; C4BPB;	1; B; B; B; 1; B; 1; 2; 1;	27039259; 33224887; 31002129; 31086659; 31049165; 28592880; 33506022; 25860442; 30453999;

	CAMP; CD58; CDA;	1; 2; 2;	27832772; 22403726; 28977491;
	CEACAM1; CYFIP1; DCTN1;	B; B; B;	24931667; 29572294; 33198314;
	DNAJC13; DPP7; DYNC1I2;	2; B; B;	33338880; 21653254; 31516392;
	EIF4G1; ENPP4; FASN;	B; B; 2;	18427574; 27259239; 25947066;
	FLNB; GAA; HRNR;	B; B; 1;	30764547; 8660303; 25614008;
	IFI16; IQGAP2; ITGB2;	2; B; B;	28415616; 28792442; 32290321;
	LAMP2; LGALS9; LRRFIP1;	B; 1; B;	27627761; 29389859; 26224133;
	MLEC; NCSTN; NUP205;	2; 1; 2;	28265552; 25887147; 32467671;
	PDAP1; PLA2G6; PPP3R1;	B; 2; B;	24124450; 28956850; 24260486;
	PSMA3; PSMA6; PSMB7;	B; B; B;	29304754; 31086659; 24758163;
	PSMD10; PSMD3; PSMD6;	2; B; 2;	36028821; 33193699; 26291246;
	PSMD8; PTPRJ; RAB3D;	2; 1; 1;	24889965; 32201537; 22259059;
	RBBP6; RHOG; SDC1;	2; 2; 1;	23799110; 28202690; 29340066;
	SEC23B; SPTBN2; STX3;	B; 2; 1;	32123160; 31049165; 27259239;
	TCN1; TCOF1; TXLNA;	1; 2; 2;	32686693; 28923911; 33075095;
	UBA52; USP7; VAMP8	B; 2; B	27270322; 32850836; 30555541
Metabolism	ACADM; ADRM1; AGL;	1; 2; 2;	29304754; 23377018; 33224887;
	AGPS; AK2; ALDH4A1;	B; B; 2;	26322624; 30588094; 32894095;
	ASXL3; ATP5F1; BDH2;	2; B; 2;	25484917; 26735889; 31819198;
	BPNT1; CDA; COX5B;	1; 2; B;	33161837; 28977491; 26869356;
	CPT2; DARS; DHRS7B;	B; B; 2;	31769430; 26421711; 30979825;
	DYNC1I2; ENPP1; EPB41L3;	B; 2; 2;	31516392; 30755817; 20651987;
	FARSB; FASN; FHL2;	1; 2; 2;	26421711; 25947066; 27415427;
	FLNB; G6PD; GAA;	B; B; B;	30764547; 27861141; 8660303;
	GALE; GCAT; GDA;	B; 2; 1;	31272438; 26627478; 31704733;
	GGT1; GLTP; GPX4;	1; B; 2;	27381639; 30271202; 31527591;
	HSD17B4; IARS; LARS;	B; 2; 1;	32678070; 31887549; 26421711;
	LDHC; LHPP; LYPLA1;	2; B; B;	31932876; 26627478; 33413672;
	MAK16; MARS; MT-ATP8;	2; B; B;	32828126; 16569247; 28814324;
	NDUFA10; NDUFAB1; NDUFV2;	1; B; 1;	26869356; 33415077; 24758163;
	NEXN; NNT; NUP205;	2; B; 2	29721183; 26454069; 32467671;
	OSBP; PBDC1; PCCB;	2; 1; B;	25729885; 25428507; 26627478;
	PIP4K2A; PITPNB; PLA2G6;	1; 2; 2;	28637795; 20515954; 28956850;
	PMPCB; PNPLA6; PSMA3;	2; 2; B;	21622897; 26627478; 29304754;
	PSMA6; PSMB7; PSMD10;	B; B; 2;	31086659; 24758163; 36028821;
	PSMD3; PSMD6; PSMD8;	B; 2; 2;	33193699; 26291246; 24889965;
	PTPLAD1; PYCR1; RPL14;	B; 2; 1;	26431493; 29805774; 28264936;
	RPL15; RPL17; RPL18A;	1; B; B;	26892688; 26597576; 30678426;
	RPL19; RPL23A; RPL24;	B; B; B;	24950402; 28147343; 30650663;
	RPL3; RPL32; RPL34;	B; B; 1;	29530001; 25864709; 29530001;
	RPL35; RPL35A; RPL36;	1; B; B;	29530001; 29530001; 29530001;
	RPS11; RPS13; RPS21;	B; B; B;	32395510; 29530001; 27233751;
	RPS23; RPS27; RPS28;	B; B; B;	30061686; 28923911; 31350888;
	SCP2; SDC1; SDC2;	B; 1; B;	31519191; 29340066; 26293675;
	SGPL1; SGSH; SQRDL;	2; 2; 2;	31049165; 31746421; 19657234;
	SUMF2; TCN1; THRAP3;	B; 1; 2;	3229626; 32686693; 29112714;
	TXNRD1; UBA52; UGP2;	1; B; B;	31814764; 27270322; 26379393;
	UQCRH; USP7; USP9X	B; 2; 2	31497032; 32850836; 28103826
Metabolism of protein	ADRM1; ARCN1; C19orf10;	2; 1; B;	23377018; 22585861; 22098948;
	CETN2; CPM; DNAJB11;	B; 1; B;	30375513; 28186967; 21423607;
	DYNC1I2; EIF2S2; EIF2S3;	B; B; B;	31516392; 17989776; 29320577;

	EIF3A; EIF3C; EIF3L; EIF4B; EIF4G1; FARSB; GMDS; GOLGB1; IARS; KDELR1; MARS; NUP205; PDIA5; PFDN1; PFDN2; PMPCB; PSMD3; PSMD6; PSMD8; RPL14; RPL15; RPS11; RPS13; RPS21; RPS23; RPS27; RPS28; SDC2; SPCS2; SPTBN2; SRP72; SRPRB; TARS; TBCA; TBCD; TMED3; UBA52; USO1	B; B; B; B; B; 1; B; 2; 2; 1; B; 2; 2; 1; B; 2; B; 2; 2; 1; 1; B; B; B; B; B; B; B; 1; 2; 1; 2; 2; B; 2; 2; B; B	29581788; 33000238; 33550985; 25611378; 18427574; 26421711; 31337362; 20685852; 31887549; 30678426; 16569247; 32467671; 26627478; 32670816; 32670816; 21622897; 33193699; 26291246; 24889965; 28264936; 26892688; 32395510; 29530001; 27233751; 30061686; 28923911; 31350888; 26293675; 27648361; 28793054; 27913494; 26421711; 33266490; 21622897; 32670816; 29085821; 27270322; 28969018
Metabolism of RNA	CNOT2; CNOT3; DDX21; DKC1; EIF4B; EIF4G1; FUS; GEMIN5; GNL3; HNRNPUL1; MT-ATP8; NHP2; NUP205; PABPN1; PRPF40A; PRPF6; PSMA3; PSMA6; PSMB7; PSMD10; PSMD3; PSMD6; PSMD8; PUF60; RPL14; RPL15; RPL17; RPL18A; RPL19; RPL23A; RPL24; RPL3; RPL32; RPL34; RPL35; RPL35A; RPL36; RPS11; RPS13; RPS21; RPS23; RPS27; RPS28; SF3A1; SF3B2; SF3B4; SNRNP70; SRRM2; SRSF10; SRSF6; UBA52	2; 2; 2; 2; B; B; B; 2; 2; B; B; 2; 2; 2; B; 2; B; B; B; 2; B; 2; 2; 2; 1; 1; B; B; B; B; B; B; B; 1; 1; B; B; B; B; B; B; B; 1; 2; 2; B; B; 2; 1; B; B	30108113; 30650663; 33328538; 31857720; 25611378; 18427574; 33000238; 28786813; 28849076; 32106407; 28814324; 28289249; 32467671; 30787966; 28786813; 32745318; 29304754; 31086659; 24758163; 36028821; 33193699; 26291246; 24889965; 33194730; 28264936; 26892688; 26597576; 30678426; 24950402; 28147343; 30650663; 30105457; 25864709; 29530001; 29530001; 29530001; 29530001; 32395510; 29530001; 27233751; 30061686; 28923911; 31350888; 26079486; 31348270; 33563334; 31788113; 27530828; 29429992; 32901876; 27270322
Muscle contraction	ATP1B3; ATP2B1; MYL12B	1; B; B	24236063; 29285225; 25029196
Neuronal system	DCTN1; EPB41L3; FLNB	B; 2; B	33198314; 20651987; 29682400
Organelle biogenesis and maintenance	ATP5F1; CETN2; DCTN1; DYNC1I2; HAUS4; MT-ATP8; PAFAH1B1; PCNT; USP9X; VWA8	B; B; B; B; 2; B; B; 2; 2; 2	26735889; 30375513; 33198314; 31516392; 26121572; 28814324; 31925297; 28426876; 28103826; 30546434
Programmed cell death	ACIN1; PPP3R1; PSMA3; PSMA6; PSMB7; PSMD3; PSMD6; PSMD8; PSMD10; UBA52	2; B; B; B; B; B; 2; 2; 2; B	24493827; 24260486; 29304754; 31086659; 24758163; 33193699; 26291246; 24889965; 36028821; 27270322
Protein localisation	ABCD3; AGPS; CHCHD2; COA6; HSD17B4; PEX19; PITRM1; PMPCB; PREP; SCP2; SLC25A4; UBA52; USP9X	1; B; 2; B; B; B; 2; 2; 1; B; B; B; 2	25004396; 26322624; 31036064; 33415077; 32678070; 29746593; 25886033; 21622897; 29651130; 31519191; 26091520; 27270322; 28103826
Reproduction	SCP2	B	31519191
Signal transduction	ABCC6; APOC1; ATP6V1A; COL5A3; CYFIP1; DYNC1I2; ECE1; EIF4B; EIF4G1; FASN; FLNB; FUS; G3BP2; IQGAP2; KHDRBS1;	2; 1; B; 2; B; B; 1; B; B; 2; B; B; B; B; B;	31565477; 30732558; 28592880; 27597234; 29572294; 31516392; 33575255; 25611378; 18427574; 25947066; 31665330; 33000238; 30586414; 28792442; 29023197;

	KIDINS220; LAMA3; LYPLA1; METAP2; MYL12B; NCSTN; PAFAH1B1; PIP4K2A; PPP1R12A; PPP1R12C; PPP3R1; PSMA3; PSMA6; PSMB7; PSMD10; PSMD3; PSMD6; PSMD8; PTPRJ; PTPRK; RCC2; RGS10; RHOG; RPS27; SDC1; SDC2; SH3GL1; SPTBN2; UBA52; USP7; USP9X	2; 2; B; B; B; 1; B; 1; 2; 2; B; B; B; B; 2; B; 2; 2; 1; 2; 2; 1; 2; B; 1; B; B; 2; B; 2; 2	31516392; 32555663; 33413672; 28588753; 25029196; 25887147; 31925297; 28637795; 26113782; 30532555; 24260486; 29304754; 31086659; 24758163; 36028821; 33193699; 26291246; 24889965; 32201537; 28731148; 28869598; 24475290; 28202690; 28923911; 29340066; 26293675; 30246154; 28793054; 27270322; 32850836; 28103826
Transport of small molecules	ABCC6; ABCD3; ABCF1; ADD3; APOC1; ATP1B3; ATP2B1; ATP6V1A; CYB5R1; EIF2S2; EIF2S3; EIF4B; FLNB; HDLBP; MYO5B; OSTM1; PEX19; PMPCB; PNPLA6; PSMA3; PSMA6; PSMB7; PSMD10; PSMD3; PSMD6; PSMD8; RPL3; SLC12A2; SLC25A4; UBA52	2; 1; 2; 1; 1; 1; B; B; 2; B; B; B; B; B; 2; 2; B; 2; 2; B; B; B; 2; B; 2; 2; B; 1; B; B	31565477; 25004396; 26374197; 31814885; 30732558; 24236063; 29285225; 28592880; 27120783; 17989776; 29320577; 25611378; 29682400; 31213659; 32670437; 28927463; 29746593; 21622897; 26627478; 29304754; 31086659; 24758163; 36028821; 33193699; 26291246; 24889965; 29530001; 19294005; 26091520; 27270322
Vesicle mediated transport	AP1G1; ARCN1; CHMP5; CLINT1; CTAGE5; DCTN1; DNASE2; DYNC1I2; GOLGB1; KDELR1; M6PR; MIA3; PAFAH1B1; PAFAH1B3; PLA2G6; RAB6B; SH3GL1; SPECC1L; SPTBN2; SSC5D; TCOF1; TGOLN2; TMED3; TPD52L1; UBA52; USO1; VAMP8	B; 1; B; 1; 2; B; 2; B; 2; 1; B; 2; B; B; 2; B; B; 2; 2; 2; 2; B; 2; B; B; B; B	31002129; 22585861; 30678426; 22613733; 26627478; 33198314; 18593951; 31516392; 20685852; 30678426; 26610586; 31929798; 31925297; 29304754; 28956850; 23442798; 30246154; 25204415; 28793054; 29854292; 28923911; 27259239; 29085821; 30678426; 27270322; 28969018; 30555541
Found by GO pathway analysis but missing from Reactome® network analysis	PFKP; AEBP1; AKAP17A; ALPPL2; ANP32E; AP3D1; ARL6IP4; ARMCX3; BTF3L4; C11orf57; C1orf210; CASC4; CCDC9; CEACAM8; CDV3; CELF2; CENPV; CISD1; COMMD8; CRELD1; CROCC; DDRGK1; DENR; DHX57; DMBT1; DNAJC10; EEF1D; EIF5B; ELMO3; EPS8L2; ERGIC1; FAM114A1; FAM3D; FAM98A; FLYWCH2; FNDC1; FYCO1; G3BP1; GALNT7; GOLGA3; GOLM1; GORASP2; GPATCH8; HHIPL2; HID1; HMGN3; HMGN4; HMGN5; HSPE1; HTATSF1; IGHV-III; IKBIP; ITFG1; KIAA1217; LRPAP1; LUC7L2; LZIC; MAP1A; MAP4; MARCKSL1; MGA1; MMRN2; MXRA7;	B; 2; 2; 1; B; 2; 2; 2; B; 2; 1; 1; 2; B; 2; 2; 2; B; 1; 2; B; 2; B; 2; B; 2; 2; 2; 2; 2; 1; 1; 1; 2; 2; 2; 2; B; 1; B; B; 1; 2; 2; 2; 2; 2; 2; B; B; B; B; 1; 2; B; 2; B; 2; 2; B; 2; B; B;	27049827; 28562334; 25394645; 31687420; 32899298; 25109897; 25886033; 29335024; 27519173; 19546167; 32399279; 27530828; 25886033; 29110374; 26421711; 23795348; 29130934; 28095368; 30280037; 26627478; 33194730; 30906477; 30224410; 29571074; 17525270; 33338880; 26735889; 31817792; 30236127; 22783413; 17555561; 31143935; 30045992; 31049165; 31423159; 30402143; 26208877; 31560169; 22399294; 29745428; 30556321; 31086659; 29125844; 33431054; 27891540; 27604143; 19292913; 26290438; 17989776; 27259239; 20461121; 33509747; 26627478; 29698419; 32939876; 33550985; 31432149; 22697792; 32823874; 32582275; 8620511; 32439908; 27817035;

	MYH4; NAP1L1; NAP1L5;	2; B; B;	30670742; 32290321; 19439102;
	NARS; NCLN; NOLC1;	1; 2; B;	21134280; 32139907; 31941945;
	NUCB1; OCIAD2; OGFR;	B; B; 2;	30280037; 28911005; 28676692;
	PABPC1L; PAGE4; PCNP;	1; 2; B;	31607220; 24708576; 22022533;
	PIN4; PLS1; POC1B-GALNT4;	2; B; 1;	23209772; 23209772; 28187432;
	PPIG; PRPF4B; PRPSAP1;	2; 2; 2;	27135912; 32033228; 23634293;
	PRR14L; PRRC2C; PTMA;	2; 2; B;	29221110; 30116770; 26517516;
	RASD2; RBM25; RBM26;	2; 2; 2;	31704733; 29693319; 25780673;
	RCN2; RCSD1; RER1;	1; 2; 2;	29218241; 28814324; 30108113;
	RERE; RIMS2; RNPEP;	2; 2; B;	25352341; 30692208; 25888189;
	S100A13; S100A6; SAFB;	2; 2; B;	30558666; 19506550; 19506550;
	SDF4; SSR4; ST13P5;	1; 1; B;	25733820; 27648361; 24817948;
	TM9SF2; TM9SF3; TMA7;	B; 1; 2;	25886033; 25886033; 28486536;
	TOR1AIP2; TPBG; TPPP3;	2; B; B;	27186291; 27323861; 33384952;
	TWF2; TXNDC17; XPNPEP1;	B; 2; B;	32860342; 31814764; 30894686;
	ZC3H18; ZG16B; ZNF428	2; 1; 2	31945087; 33336077; 29721183

[*]The EC Type where protein expression is increased: Type 1 EC = 1, Type 2 EC = 2 and both types of EC = B.
